# Imprinted expression in cystic embryoid bodies shows an embryonic and not an extra-embryonic pattern

**DOI:** 10.1016/j.ydbio.2015.04.010

**Published:** 2015-06-15

**Authors:** Tomasz M. Kulinski, M. Rita T. Casari, Philipp M. Guenzl, Daniel Wenzel, Daniel Andergassen, Anastasiya Hladik, Paul Datlinger, Matthias Farlik, H. -Christian Theussl, Josef M. Penninger, Sylvia Knapp, Christoph Bock, Denise P. Barlow, Quanah J. Hudson

**Affiliations:** a CeMM Research Center for Molecular Medicine of the Austrian Academy of Sciences, Lazarettgasse 14, AKH-BT25.3, 1090 Vienna, Austria; b IMBA, Institute of Molecular Biotechnology of the Austrian Academy of Sciences, Dr. Bohr Gasse 3, 1030 Vienna, Austria; c Department of Medicine 1, Laboratory of Infection Biology, Medical University of Vienna, 1090 Vienna, Austria; d IMP/IMBA Transgenic Service, Institute of Molecular Pathology (IMP), Dr. Bohr Gasse 7, 1030 Vienna, Austria

**Keywords:** Genomic imprinting, Visceral yolk sac (VYS), Visceral endoderm (VE), Visceral yolk sac Endoderm (ysE), Cystic embryoid bodies (EBs), DNA methylation

## Abstract

A large subset of mammalian imprinted genes show extra-embryonic lineage (EXEL) specific imprinted expression that is restricted to placental trophectoderm lineages and to visceral yolk sac endoderm (ysE). Isolated ysE provides a homogenous *in vivo* model of a mid-gestation extra-embryonic tissue to examine the mechanism of EXEL-specific imprinted gene silencing, but an *in vitro* model of ysE to facilitate more rapid and cost-effective experiments is not available. Reports indicate that ES cells differentiated into cystic embryoid bodies (EBs) contain ysE, so here we investigate if cystic EBs model ysE imprinted expression. The imprinted expression pattern of cystic EBs is shown to resemble fetal liver and not ysE. To investigate the reason for this we characterized the methylome and transcriptome of cystic EBs in comparison to fetal liver and ysE, by whole genome bisulphite sequencing and RNA-seq. Cystic EBs show a fetal liver pattern of global hypermethylation and low expression of repeats, while ysE shows global hypomethylation and high expression of IAPEz retroviral repeats, as reported for placenta. Transcriptome analysis confirmed that cystic EBs are more similar to fetal liver than ysE and express markers of early embryonic endoderm. Genome-wide analysis shows that ysE shares epigenetic and repeat expression features with placenta. Contrary to previous reports, we show that cystic EBs do not contain ysE, but are more similar to the embryonic endoderm of fetal liver. This explains why cystic EBs reproduce the imprinted expression seen in the embryo but not that seen in the ysE.

## Highlights

• Cystic EBs model embryonic, and not extra-embryonic, imprinted expression.
• The transcriptome and methylome of yolk sac endoderm, cystic EBs, and fetal liver.
• Cystic EBs are more similar to fetal liver than to yolk sac endoderm.
• Genomic analysis shows yolk sac endoderm shares epigenetic features with placenta.

## Introduction

Genomic imprinting is an epigenetic phenomenon that leads to parental allele-specific or imprinted expression of approximately 150 mouse genes, more than 80% of which are grouped into clusters. Gamete-specific DNA methylation of an imprint control element (ICE) controls imprinted expression of the entire cluster, as has been demonstrated by genetic deletion in seven cases (Barlow and Bartolomei, 2014). In the most common mechanism of imprinted silencing, the unmethylated ICE allele is associated with the promoter of a long non-coding (lnc) RNA that initiates silencing of imprinted genes in the cluster, as has been demonstrated for four of the seven clusters (Mancini-Dinardo et al., 2006; Meng et al., 2012; Sleutels et al., 2002; Williamson et al., 2011). While most imprinted genes show some degree of tissue-specific regulation of imprinted expression (Prickett and Oakey, 2012), two broad types have been identified: multi-lineage (ML) and extra-embryonic lineage (EXEL) specific imprinted expression. ML imprinted expression is seen in both embryonic and extra-embryonic tissues. EXEL imprinted expression is restricted to placental trophectoderm lineages and the visceral yolk sac (VYS) endoderm layer (ysE) (Hudson et al., 2011). In imprinted clusters, genes showing EXEL specific imprinted expression tend to be located further away from the ICE and the lncRNA that silences them, presenting a model of long-range *cis* silencing by lncRNAs (Kulinski et al., 2013).

Extra-embryonic tissues show a chromatin state distinct from embryonic lineages, for example displaying global DNA hypomethylation (Chapman et al., 1984; Popp et al., 2010; Rossant et al., 1986). EXEL imprinted expression has been suggested to arise from a more permissive chromatin organization in extra-embryonic tissues that allows the silencing mechanism to extend over greater distances (Hudson et al., 2010; Kulinski et al., 2013), or from repression of EXEL specific enhancers by imprinted lncRNAs (Pauler et al., 2012). Testing these hypotheses *in vivo* require laborious genetic experiments to manipulate the imprinted cluster in the endogenous locus in the mouse. In comparison, genetic studies in *ex vivo* model systems are faster and more cost effective. Research on the mechanism of ML imprinted expression has been facilitated by the use of an *ex vivo* model system employing ES cell differentiation (Kohama et al., 2012; Latos et al., 2009; Meng et al., 2012; Wood et al., 2010). The ysE, which arises from the primitive endoderm of the pre-implantation embryo, forms the external layer of the bilaminar VYS that encloses the embryo after the embryo turns around E8.5 and remains until birth. Although stem cells known as XEN cells that model the primitive endoderm have been derived and display some markers of visceral endoderm, they are largely unable to contribute to the visceral endoderm in chimeras (Kunath et al., 2005). In contrast, ES cells differentiated *in vitro* by aggregation into small non-attached cell clumps called embryoid bodies (EBs) eventually develop cystic structures that closely resemble the bilaminar structure of the VYS and have been considered to contain ysE (Abe et al., 1996; Doetschman et al., 1985; Kurosawa, 2007; Yasuda et al., 2009). Electron microscopy studies also showed the outer epithelial layer of cystic EBs contained microvilli and cytoplasmic vacuoles similar to the ysE (Doetschman et al., 1985). Further support that differentiating ES cells into cystic EBs provides an *ex vivo* model of ysE development comes from studies showing that the outer endodermal-like layer expresses visceral endoderm markers such as *Afp* and *Ttr*, as well as endoderm-specific transcription factors such as *Hnf1, vHnf1, Hnf3b, Hnf4* and *Gata4* (Abe et al., 1996; Doetschman et al., 1985; Koike et al., 2007; Kurosawa, 2007; Sajini et al., 2012; Sakai et al., 2011; Soudais et al., 1995). Despite this, the ability of cystic EBs to model EXEL imprinted expression of the ysE has not yet been tested.

Here we developed a robust protocol to differentiate mouse ES cells into cystic EBs and determined if this *ex vivo* system can be used as a model of ysE imprinted expression. We show that many ysE EXEL imprinted genes were either not expressed, or very lowly expressed in cystic EBs. Furthermore, while cystic EBs robustly displayed the ML type of imprinted expression, all EXEL genes expressed above baseline levels exhibited biallelic expression. Using whole genome bisulphite converted DNA sequencing (WGBS) we showed that cystic EBs exhibit a high level of DNA methylation comparable to that observed in fetal liver, and not the lower levels observed in ysE. Using RNA-seq transcriptome and WGBS analysis, we further showed that cystic EBs, rather than resembling ysE, are more closer related to the definitive endoderm derived fetal liver. Finally, we comprehensively annotated the ysE transcriptome and identified a characteristic set of genes that are absent from the cystic EB transcriptome. Together these results indicate that cystic EBs have an embryonic-like epigenetic state, contain embryonic endoderm rather than extra-embryonic endoderm, and, while a useful resource for analyzing ML imprinted expression in different tissue-types, they cannot be used as a model for EXEL imprinted expression.

## Material and methods

### Tissue collection and VYS layers separation

Mice were bred and housed at the IMBA/IMP facility in Vienna in strict accordance with national recommendations under Laboratory Animal Facility Permit MA58-0375/2007/4. Fetal liver, VYS and VYS endoderm (ysE) were collected from E12.5 FVB/N or CAST/EiJ× FVB/N F1 crosses. Separated ysE and mesoderm was collected from E9.25 FVB/N embryos. In each case the entire litter was pooled for each sample. The VYS endoderm and mesoderm layers were manually separated using a DispaseII pre-treatment for 1 h (E9.5) and 2 h (E12.5) as described (Hudson et al., 2011). The VYS mesoderm was collected together with the vasculature and the basement membrane, while the ysE was collected alone. The accuracy of separation was assessed using RT-qPCR for markers of ysE (*Afp*) and mesoderm (*Flk1*).

### ES cell lines

Mouse ES cell lines were derived from CAST/EiJ×FVB/N crosses (described below, clones CF-C2 (XY) and FC-A2 (XY) were used), or from C57BL/6×129 crosses (clone A9 (XY) from Anton Wutz, clone JN (XX) from Jennifer Nichols). ES cells were maintained in DMEM based medium with 15% fetal calf serum and LIF, on irradiated E12.5 mouse embryonic fibroblast feeders according to standard protocols. ES cells were differentiated in retinoic acid (RA) as described previously (Latos et al., 2009) or into cystic EBs as described below. The A9 and JN cells showed a normal karyotype and the CF-C2 and FC-A2 clones showed normal differential methylation of tested ICE as described below. All ES cell lines also showed normal ES cell morphology and growth dynamics.

### Derivation of ES cells from CAST×FVB F1 crosses

ES cells were derived from reciprocal crosses of CAST/EiJ×FVB/N using a method adapted from a published protocol (Bryja et al., 2006). In brief, harvested blastocysts were incubated in Tyrodes acid for 30–60 s till the zona pellucida dissolved. Afterwards, blastocysts were plated onto MEF feeder layers in SR-ES medium (Table S2) and left undisturbed for two days to allow attachment. Medium was changed every two days. After 6 days the blastocysts hatched and were trypsinized (0.25% trypsin–EDTA) for the first time and plated onto a new well of MEF-feeders in ES medium (Table S2). After one additional week ES cells were growing out and the best looking colonies were sub-cloned and mechanically passaged. Between 3 and 8 ES cells sub-clones were picked per blastocyst (5 CAST×FVB blastocysts, 3 FVB×CAST blastocysts, maternal allele on the left). Each clone was sex-typed, checked for differential methylation of the *Dlk*, *Igf2r* and *Kcnq1* cluster ICE, and the general morphology and growth characteristics were noted.

### A robust protocol for cystic EB differentiation

**Fig. 1:**
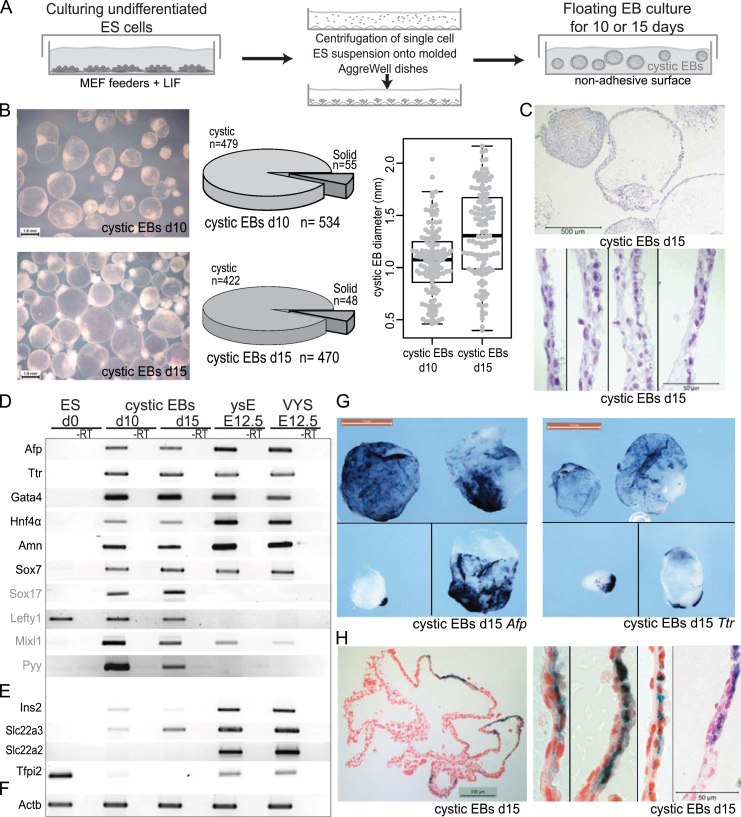
Cystic embryoid bodies (EBs) express a subset of endoderm markers and have a membrane-like morphology. (A) The protocol used to differentiate ES cells into cystic EBs. (B) Left: light microscope image of cystic EBs (scale bar: 1 mm) differentiated for 10 (d10) and 15 (d15) days. Middle: the relative abundance of cystic and solid EBs at d10 and d15. Right: the diameter of cystic EBs at d10 and d15. (C) Histological section of d15 cystic EBs stained with haematoxylin and eosin showing their characteristic organization with a solid node of cells localized to one end and an outer bi-laminar layer containing columnar epithelium (scale bar top: 500 µm, bottom: 50 µm). (D) RT-PCR shows that general endoderm markers (black font) and specific anterior and definitive endoderm markers (gray font) are robustly detected in cystic EBs, but the latter markers are not found in E12.5 visceral yolk sac endoderm (ysE) or visceral yolk sac (VYS) (ES d0: undifferentiated ES cells). (E) RT-PCR shows that four extra-embryonic lineage (EXEL) specific imprinted genes are either lowly expressed (*Slc22a3*, *Ins2*) or not detectable after 40 PCR cycles in d10/d15 cystic EBs, whereas they are robustly expressed in ysE and VYS. With the exception of *Tfpi2*, ES d0 cells also do not express the EXEL genes tested. (F) Control actin RT-PCR. (G) Whole mount *in situ* hybridization (WISH) detects the expression of endoderm markers *Afp* and *Ttr* in cystic EBs (scale: 1 mm). (H) Histological sections of WISH stained cystic EBs localize *Afp* and *Ttr* expression to the outer columnar epithelium layer (scale left: 100 µm, right: 50 µm).

To produce comparable EB populations we started differentiations from ES cells between 18 and 21 passages. Previously multiple protocols for ES cell differentiation into cystic EBs have been proposed. However, most of them depend on a random or poorly controlled aggregation of ES cells and thus are hampered by poor reproducibility of the starting aggregate size (Kurosawa, 2007). The starting cell number in the initiating clumps in suspension cultures has been shown to determine the simple EB versus cystic EB composition of the differentiated populations and substantially influence endoderm marker expression (Koike et al., 2007; Yasuda et al., 2009). To increase the reproducibility of ES cell differentiation to cystic EBs and lineage choice we used AggreWell™400 plates to control the aggregate starting size (Stemcell Technologies #05893) as previously described (Fig. 1A) (Antonchuk, 2013). We optimized the protocol to maximize the proportion of cystic EBs and determined that starting aggregates of 1000–2000 ES cells yielded consistent cystic morphology (Fig. 1B). Therefore we proceeded with the analysis of cystic EBs initiated with an average starting aggregate of 1000 cells (1.2×10^6^ cells suspended in 2 ml media centrifuged into 1200 microwells). After 8 h incubation in AggreWell™ plates in LIF+ES cell media to allow the cells to attach to each other, the aggregates were flushed out with a stream of media from a pipette and transferred to non-adhesive dishes (Corning Ultra Low Attachment 75 cm^2^ flasks) and cultured in 25–30 ml media as floating cultures in ES cell medium without LIF (25 ml media in early stages, 30 ml media from d10). By day 5 of differentiation, EBs were still very homogenous in shape and size with many showing the first signs of cavitation by developing hollow buds. After day 5, those buds developed and underwent a phase of extensive growth between day 7 and 10 to eventually form cystic EBs. By day 10 and 15 of differentiation approximately 90% of the aggregates represented cystic EBs with an average size of 1.05 mm and 1.32 mm, respectively (Fig. 1B). These structures could be sustained in culture for at least 2 months, but their morphology and size did not significantly change beyond day 20. An outer layer of columnar epithelial cells surrounded the solid EBs whereas cystic EBs usually had a pole resembling the solid EB and an additional cystic part consisting of a large membrane-like structure that had mostly a double cell layer (Fig. 1C). The outer layer of cystic EBs appeared to show an epithelial morphology whereas the inner lining resembled mesothelial cells (Fig. 1C). Between those two layers empty spaces were sometimes seen that may represent blood islands. In combination these features resemble the yolk sac histology as reported before (Fig. S1A) (Wang et al., 1992; Zhou et al., 2005). Using this protocol we were also able to differentiate cystic EBs from the classical 129/SvPas (D3) and 129/SvEv (CCE) inbred ES cell lines, which showed a similar morphology and expression of markers to the F1 ES cells. Taken together these results show that the EB differentiation protocol that we applied, allows robust generation of a relatively pure population of cystic EBs reproducing the previously reported morphology (Abe et al., 1996; Doetschman et al., 1985; Kurosawa, 2007; Niwa, 2010; Yasuda et al., 2009).

### Histology and whole mount *in situ* hybridizations (WISH)

To assess cystic EB histology, cystic EBs differentiated for 10 (d10) and 15 (d15) days were fixed for 10 min in 4% paraformaldehyde, cryopreserved in 30% sucrose in PBS overnight at 4 °C, and snap-frozen in OCT and cryosectioned using CryoJane® Tape-Transfer System. Subsequent haematoxylin–eosin staining was performed according to standard protocol. Probe sequences for WISH were PCR amplified (primers in Table S3), cloned into pGEM-T® easy vector and then PCR amplified using M13 primers. This purified PCR product was then used as a template for T7 or SP6 polymerase *in vitro* transcription to produce antisense and sense riboprobes depending on the template orientation in the vector. Signal detection was performed using *Afp, Ttr*, *Lefty* and *Pyy* probes according to a published protocol (Piette et al., 2008). Cystic EBs d15 were pierced to avoid probe trapping. WISH samples were cryosectioned as above and counterstained with ContrastRed® and photographed using a stereoscopic microscope.

### DNA and RNA analysis

Total RNA and DNA were extracted using TRI-reagent (Sigma-Aldrich T9424) according to the manufacturers protocol. Total RNA was DNase1 treated using the DNA-free™ kit (Ambion). Reverse transcription (RT) was performed using the Revert Aid First Strand cDNA Synthesis Kit (Fermentas) with random hexamer primers. cDNA was amplified according to standard procedures using the GoTaq® Flexi DNA polymerase (Promega) or the Long PCR Mix enzyme (Fermentas). Primers are listed in Supplementary materials (Table S3). PCR amplicons were resolved on standard agarose gel and purified using Wizard® SV Gel Clean-Up System and subject to Sanger sequencing. For genes showing a reciprocal bias the allelic contribution was quantified from the sequencing chromatograms using Mutation Surveyor® software.

### RNA-seq, ChIP-seq, and whole genome bisulfite sequencing (WGBS)

Ribosomal RNA was depleted from total DNaseI-treated RNA using the RiboZero rRNA removal kit (Human/Mouse/Rat) (Epicenter). Three different methods of RNA-seq libraries preparation were used (detailed in Table S4): (1) non-stranded RNA-seq from double-stranded cDNA (Huang et al., 2011), (2) strand-specific RNA-seq libraries prepared using ScripSeq v1 kit (Epicenter), and (3) TruSeq RNA Sample Prep Kit v2 (Illumina) modified by using dUTP instead of dTTP during second-strand cDNA synthesis, followed by subsequent uracil-DNA glycosylase (UDG) (Fermentas) degradation of the second strand after adapter ligation to preserve strand information as described (Sultan et al., 2012). Native ChIP for H3K4me3 (antibody: cat. 07-473, lot 0608038479, Millipore) was conducted by homogenizing 20× VYS under liquid nitrogen (E12.5 FVB/N pooled from 2 litters) and then proceeding as previously described (Regha et al., 2007). ChIP-seq libraries were prepared using TruSeq ChIP Sample Prep Kit (Illumina). Libraries for whole genome bisulphite sequencing (WGBS) were prepared using the TruSeq DNA LT Sample Prep Kit v2 (Illumina) with a conversion step before the library amplification using the EZ DNA Methylation-DirectTM Kit reagents according to the protocol (except the bisulfite reaction mix was diluted 0.9×), with 20 cycles of 1 min at 95 °C then 10 min at 60 °C. The incubation time for desulphonation was extended to 30 min. All sequencing was performed by the Biomedical Sequencing Facility (BSF) in Vienna using the Illumina HiSeq 2000 platform (detailed in Table S4).

### Bioinformatic analysis

The RNA-seq, ChIP-seq and WGBS data was aligned using GSNAP aligner and further processed and analyzed as described below. Custom scripts, Samtools (version 0.1.16) and RSeQC (version 2.3.3) were used to generate RPKM tables, read count tables, and UCSC tracks for visualization. FastQC was used to assess sequence read quality (Quinlan and Hall, 2010; Wu and Nacu, 2010). Cufflinks (version 2.1.1) was used to *de novo* annotate transcripts in the RNA-seq data. To reduce artefacts the *de novo* Cufflinks annotations were filtered for spliced transcripts with a minimum length of 500 nt using custom scripts in R programing language (http://www.r-project.org), incorporating Bedtools (v2.17.0). Custom scripts incorporating Bedtools were also used for feature overlap analysis between the *de novo* transcripts and DESeq and DEXSeq BioConductor packages were used to call differentially expressed transcripts and differential exon usage respectively. Gene ontology analysis was done using DAVID online tools (Huang et al., 2009). ChIP-seq data was analyzed using MACS (version 1.4.2) and SeqMonc (http://www.bioinformatics.babraham.ac.uk/projects/seqmonk/) (Anders and Huber, 2010; Huang et al., 2009; Quinlan and Hall, 2010; Trapnell et al., 2010; Zhang et al., 2008). Graphs displaying the result of bioinformatic analysis were constructed in R programing language (http://www.r-project.org). Supervised hierarchical clustering of E9.25 ysE, E12.5 ysE, cystic EBs and E12.5 fetal liver samples was done by the expression of selected developmental markers using Pearson’s correlation as a measure of clustering distances and the “complete” agglomeration method for dendogram generation (Fig. 4D). Unsupervised classification of E9.25 ysE, E12.5 ysE, cystic EBs and E12.5 fetal liver samples was done according to 3133 RefSeq protein coding genes that showed a highly significant (*P*<10^−3^) difference in expression between E12.5 fetal liver and E12.5 ysE (Fig. S4E) using the “complete” agglomeration method for dendrogram generation (Fig. 4E). CpG methylation in the WGBS reads was called using the BisSNP package using low coverage settings (Liu et al., 2012) and a FVB/Cast SNP list (Keane et al., 2011; Wong et al., 2012). Custom scripts incorporating Bedtools were used to prepare the count tables of CpG methylation over given features. Hierarchical clustering of tissues by DNA methylation in Fig. 3B was done using 5 kb non-overlapping windows that showed the greatest similarity between replicates (*n*=4996), but differed with at least one other tissue. Windows were selected that had an interquartile range (IQR) <10% within tissue replicates, but IQR >20% between tissues. Euclidian distances and the “complete” agglomeration method was used for dendrogram generation. All custom scripts are available on request. High throughput sequencing data has been deposited in NCBI's Gene Expression Omnibus (GEO) database under the accession number GSE56276 (http://www.ncbi.nlm.nih.gov/geo/query/acc.cgi?acc=GSE56276) (Table S4).

## Results

### Cystic EBs express endoderm markers but lack expression of some EXEL imprinted genes

We developed a protocol to differentiate ES cells into a relatively pure population of cystic EBs (approximately 90% of EBs after 10 or 15 days of differentiation) that reproduced the previously published cystic morphology more robustly (Fig. 1A–C) (Doetschman et al., 1985; Kurosawa, 2007; Niwa, 2010; Yasuda et al., 2009). Cystic EBs were reported to express endoderm markers in their outer epithelial layer (Abe et al., 1996; Koike et al., 2007; Yasuda et al., 2009). We were able to confirm expression of endoderm markers in our cystic EBs by RT-PCR, including *Afp* and *Ttr* as well as the *Gata4* and *Hnf4α* transcription factors that are required for endoderm development (Fig. 1D). As positive controls we used isolated ysE dissected free of the mesodermal layer and blood islands (Hudson et al., 2011) and intact VYS from E12.5 embryos. We also confirmed that *Afp* and *Ttr* expression is localized to the external epithelial cell layer of cystic EBs by *in situ* hybridizations for these genes (Figs. 1G–H and S1B,C). RT-PCR detected expression of *Amn* and *Sox7* in cystic EBs and in ysE and VYS (Fig. 1D). Both genes are expressed in the primitive endoderm derived visceral endoderm (VE). However, by E7.5 *Sox7* localizes mainly to the extra-embryonic part of VE that overlays the extra-embryonic ectoderm and later develops into ysE (Kanai-Azuma et al., 2002), *Amn* localizes to both the extra-embryonic VE and the embryonic VE that overlays the epiblast and later contributes to embryonic endoderm (Kalantry et al., 2001; Kwon et al., 2008). Later in development *Sox7* is also expressed in developing gut, pancreas, liver and lung, and during embryonic angiogenesis and in heart (Lioubinski et al., 2003; Matsui et al., 2006; Takash et al., 2001), whereas *Amn* plays an essential role for intestine and kidney proximal tubules development and is expressed in those tissues (Strope et al., 2004). Therefore, these genes are not specific markers for extra-embryonic VE and ysE. The expression of *Sox17* was clearly detected in cystic EBs but not in ysE (Fig. 1D). Based on the known expression pattern of *Sox17*, this indicates that cystic EBs either contain an earlier developmental stage of VE prior to VYS formation or contain definitive endoderm (Kanai-Azuma et al., 2002). We also examined *Lefty1* and *Mixl1* that are markers of embryonic VE and definitive endoderm, although *Mixl1* is also expressed in other lineages including mesoderm (Pearce and Evans, 1999). *Lefty1* was detected in cystic EBs and undifferentiated ES cells but not in ysE (Figs. 1D and S1D). *Mixl1* was expressed in cystic EBs but was also detected in ysE (Fig. 1D). *Pyy*, which has been reported to have its expression restricted to foregut definitive endoderm derivatives (Hou et al., 2007), was detected in cystic EBs but not in ysE (Figs. 1D and S1E). We then tested the expression of four EXEL imprinted genes (Fig. 1E). All were robustly expressed in ysE and VYS samples, but only after 40 cycles of PCR was low level expression of *Ins2* and *Slc22a3* detected in cystic EBs, while *Slc22a2* and *Tfpi2* were not detected. *Tfpi2* was strongly expressed in undifferentiated ES cells prior to down regulation upon differentiation in EBs. We also examined marker expression by PCR at day 5 of differentiation prior to cystic EB formation when EBs were still very homogenous in shape and size but many were beginning to show the first signs of cavitation with developing hollow buds. *Afp*, *Ttr* and *Slc22a3* were already detected at this early stage, but the specific ysE marker *Slc22a2* was not detected (Fig. S1F). In summary, cystic EBs express markers of endoderm in their outer epithelial layer as reported before, but the expression pattern of a limited set of markers indicates a definitive endoderm rather than an extra embryonic ysE identity. Furthermore, cystic EBs fail to express, or show very low expression, of some EXEL imprinted genes that are typically highly expressed in the ysE.

### Cystic EBs show ML imprinted expression

**Fig. 2:**
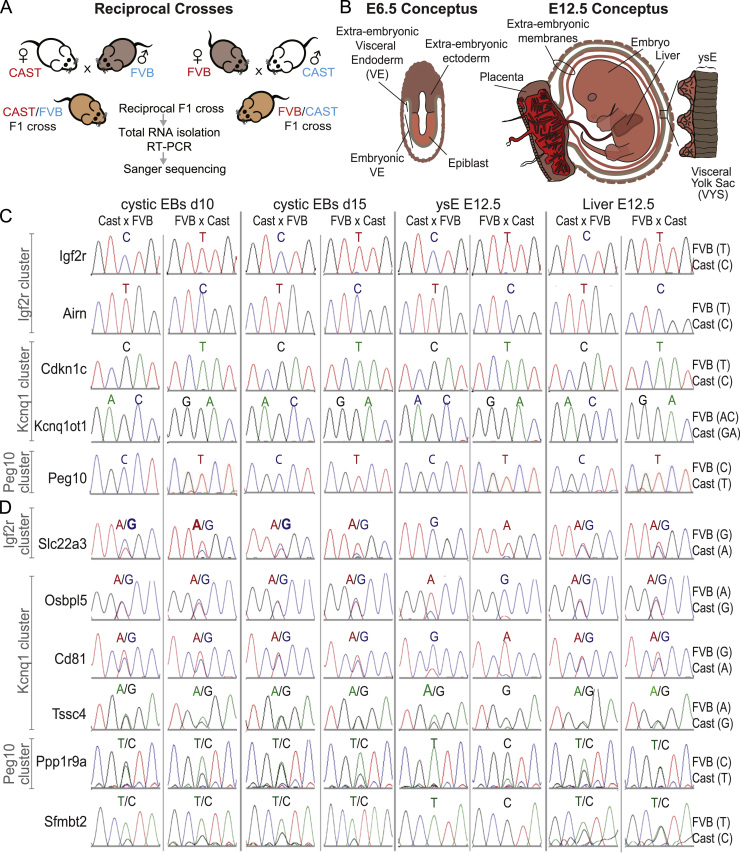
Cystic EBs show multi-lineage (ML) but not extra-embryonic lineage (EXEL) specific imprinted gene expression. (A) The breeding scheme used to obtain reciprocal crosses between the FVB/N (FVB) and CAST/EiJ (CAST) mouse strains for imprinted expression analysis using single nucleotide polymorphisms (SNPs). (B) Embryos at E6.5 and E12.5 indicating embryonic and extra-embryonic tissues (VYS diagram adapted from Hudson et al. (2011)). (C) The ML imprinted genes *Igf2r*, Cdkn1c (maternally-expressed), and *Kcnq1ot1, Airn*, *Peg10* (paternally-expressed) that represent 3 out of 4 imprinted clusters that contain EXEL genes, show the expected reciprocal imprinted expression pattern in cystic EBs, E12.5 yolk sac endoderm (ysE) and E12.5 fetal liver. The expressed SNP is shown above each Sanger sequencing chromatogram, FVB and CAST alleles are indicated on the right. (D) The EXEL genes *Osbpl5*, *Cd81*, *Tssc4*, and *Ppp1r9a* from the *Kcnq1* and *Peg10* imprinted clusters show biallelic expression in cystic EBs. The solo EXEL gene *Sfmbt2* shows a strain bias between FVB and CAST so biallelic expression in cystic EBs was validated in B6/129 ES cells (Fig. S2B). The *Slc22a3* EXEL gene from the *Igf2r* cluster shows a reciprocal bias in cystic EBs with very low expression requiring 40 PCR cycles and multiple reactions to acquire enough DNA for sequencing. Each EXEL gene shows imprinted expression in E12.5 ysE and biallelic expression in E12.5 fetal liver. SNPs are displayed above the chromatograms with a single base indicating robust imprinted expression, both SNP bases indicating biallelic expression, and both SNP bases with one in bolded font indicating biased expression. The maternal allele is written on the left.

We next assessed the ability of cystic EBs to model imprinted expression. We used RT-PCR followed by Sanger sequencing of cystic EBs differentiated from two F1 ES cell lines with a FVB/CAST and the reciprocal CAST/FVB genetic background, plus E12.5 ysE and fetal liver from the same crosses (Fig. 2A, B). We first examined allelic expression of a selection of ML imprinted genes that belong to clusters that also contain EXEL imprinted genes expressed in cystic EBs. All tested ML genes showed the expected maternal (*Igf2r, Cdkn1*), or paternal (*Airn, Kcnq1ot1, Peg10*), imprinted expression pattern in cystic EBs and in the extra-embryonic (ysE) and embryonic (fetal liver) endoderm controls (Fig. 2C). *Igf2* only showed a paternal expression bias (30% maternal and 70% paternal) in cystic EBs (Fig. S2A). The incomplete imprinted silencing of *Igf2* may be explained by the presence of early haematopoiesis in cystic EBs (Dang et al., 2002; Doetschman et al., 1985) as human hematopoietic cells relax *Igf2* imprinted expression (Hofmann et al., 2002). Together these results demonstrated that imprinted expression is regulated normally in cystic EBs. Thus cystic EBs are a useful developmental model of ML imprinted expression.

### Cystic EBs do not reproduce the EXEL imprinted expression of yolk sac endoderm

The lack of expression of some ysE EXEL imprinted genes in cystic EBs (Fig. 1D), demonstrated that cystic EBs could not be used to model EXEL-specific imprinted expression for these genes. To investigate the utility of cystic EBs in general to model EXEL imprinted expression we examined imprinted expression of ysE EXEL genes that were expressed in cystic EBs. The EXEL imprinted genes *Osbpl5*, *Cd81*, *Tssc4*, *Sfmbt2* and *Ppp1r9a* showed biallelic expression in cystic EBs similar to that observed in fetal liver, while the ysE showed imprinted expression (or strong maternal bias in the case of *Tssc4*). *Sfmbt2* showed a strain bias towards the FVB allele in cystic EBs and fetal liver although there was clear imprinted expression in ysE (Fig. 2D), so we confirmed the biallelic expression of *Sfmbt2* in cystic EBs derived from two independent B6/129 F1 ES cell lines (Fig. S2B). The reported ysE EXEL gene *Pon2* could not be tested in the CAST/FVB system due to a strong strain bias, but it also showed biallelic expression in cystic EBs derived from the two B6/129 F1 ES cell lines (Fig. S2B). In contrast to other EXEL imprinted genes, the lowly expressed *Slc22a3* demonstrated a reciprocal maternal biased expression of approximately 66% maternal to 34% paternal in d10 cystic EBs and 70% maternal to 30% paternal in d15 cystic EBs, while the control ysE showed 100% maternal expression (Figs. 2D and S2C). At d5 the low level of *Slc22a3* detected showed biallelic expression further indicating that no ysE is present at this early stage of cystic EB differentiation (Fig. S2D). ES cells differentiated in a monolayer with retinoic acid (RA) do not contain any extra-embryonic tissue type, but showed a similar low level of *Slc22a3* expression and maternal bias to d10 and d15 cystic EBs, indicating that at least in cell culture, *Slc22a3* may show maternal biased expression not restricted to EXEL tissues (Fig. S2E). Together these results show that cystic EBs do not display the EXEL imprinted expression pattern typical of ysE, but instead show an imprinted expression pattern resembling that seen in embryonic lineages.

### Cystic EBs lack the epigenetic hallmarks of yolk sac endoderm

**Fig. 3:**
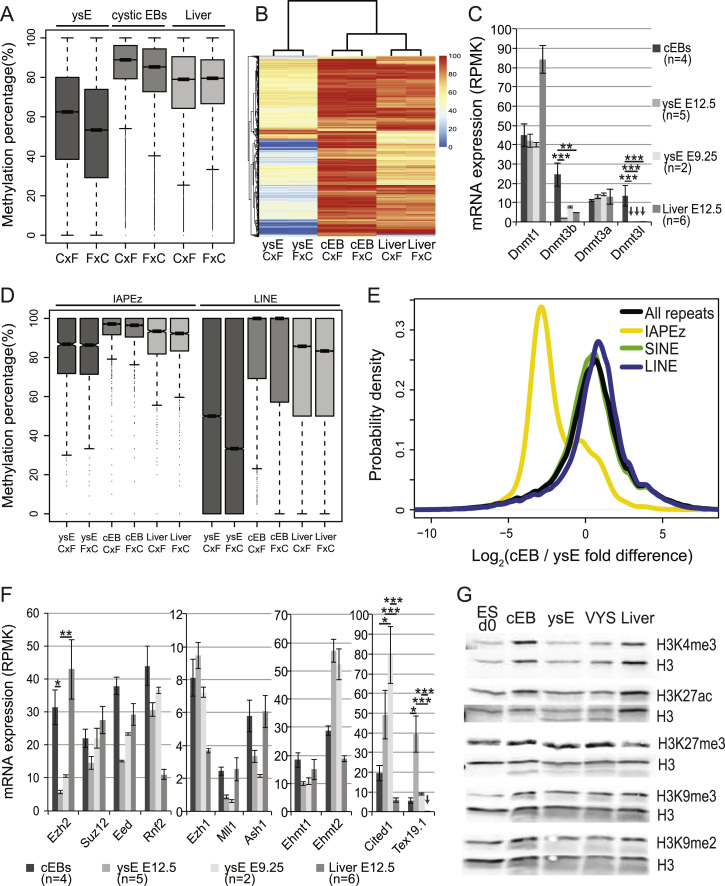
Cystic EBs lack epigenetic features of visceral yolk sac endoderm. (A) Global DNA methylation in E12.5 yolk sac endoderm (ysE) is lower than in d15 cystic EBs, which shows greater similarity with the DNA methylation level of E12.5 fetal liver. Whole genome bisulfite sequencing (WGBS) data was analyzed using 5 kb windows across the genome. Box plots show the range of DNA methylation for each tissue indicating the median and the interquartile range (IQR). (B) Cystic EBs (d15) cluster closer to E12.5 fetal liver than E12.5 ysE by DNA methylation levels. Hierarchical clustering was done using 5 kb windows (*n*=4996) that showed the greatest similarity between replicates, but differed with at least one other tissue (details in Supplementary materials). (C) *De novo* DNA methyltransferases *Dnmt3b* and *Dnmt3l* are more highly expressed (reads per kilobase per million reads (RPKM)) in RNA-seq data from cystic EBs than in E9.5, 12.5 ysE and fetal liver. (D) IAPEz repeats are highly methylated, but show a lower level of DNA methylation in E12.5 ysE compared to d15 cystic EBs, which show levels more similar to E12.5 fetal liver. LINE repeats are less methylated, but show a similar pattern with E12.5 ysE showing a lower level of DNA methylation than d15 cystic EBs, which show levels more similar to fetal liver. Box plots (shaded as in 3A) show the percentage of methylation for each sample indicting the median and IQR. (E) E12.5 ysE expresses IAPez repeats at a higher level than cystic EBs, while SINEs, LINEs, and all repeats show no difference in expression. The Kernel density plot displays the probability of repeats showing different expression in these two tissues (*x*-axis: log_2_ fold difference in expression, negative values represent overexpression in E12.5 ysE, positive values represent overexpression in cystic EBs). (F) The expression of selected genes from chromatin modifying complexes from RNA-seq data. (G) No difference in the global level of histone modifications was detected by Western blot analysis for H3K4me3, H3K27ac, H3K27me3, H3K9me3 and H3K9me2 histone modifications in undifferentiated ES cells, cystic EBs d15, E12.5 ysE, E12.5 total VYS and E12.5 fetal liver. A pan-H3 antibody is provided below each of the modifications as a loading control. Error bars in (C) and (F) show the standard error of the mean (*P≤0.05, **P≤0.01, and ***P≤0.001).

Cystic EBs lack EXEL imprinted expression, a characteristic epigenetic feature of ysE. Therefore, we investigated cystic EBs for other epigenetic features that are known to distinguish the extra-embryonic ysE from embryonic lineages. The extra-embryonic lineages have been shown to be hypomethylated on specific repetitive elements and genic sequences (Chapman et al., 1984; Rossant et al., 1986) and placenta globally shows low DNA methylation (Popp et al., 2010). To determine the DNA methylation state in cystic EBs we performed WGBS on d15 cystic EBs and compared it to E12.5 ysE and fetal liver. Fig. 3A shows that genome-wide ysE has relatively low levels of DNA methylation (median 53.3–62.5%) compared to fetal liver (median 79–79.6%) consistent with previous findings comparing the extra-embryonic placenta and embryonic tissues (Popp et al., 2010). However, DNA methylation in cystic EBs does not resemble ysE, but displays a slightly higher level than fetal liver (median 85.3–89.9%). Moreover by hierarchical clustering the global methylation pattern of cystic EBs was closer to fetal liver than to ysE (Fig. 3B). A similar picture was observed when tissues were clustered by the methylation of repeats, promoters or CpG islands (Fig. S3A–C). Analysis of RNA-seq data showed that the maintenance DNA methyltransferase *Dnmt1* was expressed at a high level in all tissues, while the *de novo* methyltransferase *Dnmt3a* was expressed at a more moderate level in all tissues (Fig. 3C). The *de novo* methyltransferases *Dnmt3b* and *Dnmt3L* were expressed more highly in cystic EBs compared to E12.5 and E9.5 ysE and E12.5 liver. This may explain why a slightly higher level of global DNA methylation was seen in cystic EBs compared to fetal liver. However, analysis of the pluripotency markers *Oct4* and *Rex1* showed that although they are strongly down regulated during cystic EB differentiation, they are still expressed, raising the possibility that remnant pluripotent cells may exist in cystic EBs (Fig. S1G). This could explain the expression of *Dnmt3l* in cystic EBs, as it showed a similar pattern of expression to the pluripotency markers during cystic EB differentiation, but could not explain the expression pattern of *Dnmt3b*, which was expressed at a higher level than the pluripotency markers (Fig. S1H). High *Dnmt3b* expression has been previously reported in EBs (Leitch et al., 2013), and may be explained by the presence of early embryonic ectoderm cells in EBs, which express a high level of *Dnmt3b* in the early post-implantation embryo (Okano et al., 1999).

We next evaluated levels of methylation on CpG islands (Fig. S3D), genic elements (Fig. S3E), and different types of repeats (Figs. 3D and S3F,G). As expected the bulk of CpG islands were unmethylated (median <10%) in contrast to the rest of the genome, which was highly methylated in fetal liver and cystic EBs (median 79–89.5%), and showed intermediate levels in ysE (median 53.3–62.5%) (Fig. S3D). Promoters showed a lower level of DNA methylation than other genic elements, which was expected due to the overlap of many CpG islands with promoters (Fig. S3E), and was also consistent with previous reports (Lister et al., 2009; Popp et al., 2010). Protein-coding exons, introns and UTRs displayed a similar level of methylation within a tissue type, showing a high level for fetal liver and cystic EBs (>80%) and a more moderate level for ysE (median 65.8–73.7%, Fig. S3E). Repeats were highly methylated in cystic EBs and fetal liver, but generally less methylated in ysE (median 50–70%) (Fig. S3F). This pattern was similar for the SINE, LTR, satellite and DNA repeat families (Fig. S3G). The intracisternal A particle (IAP) IAPEz family of retrotransposons showed a higher level of DNA methylation than other repeats, although ysE still showed a lower level of methylation than cystic EBs and fetal liver. In contrast, LINEs were less methylated than other repeats in ysE (median 30–50%), but not in cystic EBs and fetal liver (Fig. 3D). It has previously been shown that IAPEz repeats are highly expressed in the extra-embryonic placenta, but not in embryonic tissues (Reichmann et al., 2013). Analysis of RNA-seq data showed that ysE express IAPEz repeats highly compared to cystic EBs (Fig. 3E) and fetal liver (Fig. S3H). SINEs, LINEs and all repeats grouped together showed no difference in expression between the tissues. IAPEz expression in cystic EBs, fetal liver and VYS mesoderm was similar (Fig. S3I, J). Thus ysE shows high expression of IAPEz like placenta, while cystic EBs show low IAPEz expression similar to epiblast-derived lineages. LINEs are not expressed in ysE despite low levels of DNA methylation. In hypomethylated primordial germ cells genome defense genes repress the expression of retrotransposable elements. Similarly, in placenta LINEs are lowly methylation and are suppressed by the genome defense gene TEX19.1, which has been reported to show expression restricted to germ cells and the extra-embryonic placenta (Reichmann et al., 2013). *Tex19.1* is highly expressed in E12.5 ysE and moderately expressed in E9.5 ysE and cystic EBs, but not expressed in E12.5 liver (Fig. 3F). This indicates that expression of hypomethylated LINEs in ysE may be suppressed by TEX19.1, as occurs in placenta, although the expression levels indicate that this may be more important in late rather than early ysE.

The repressive histone modifying complex PRC2, which deposits H3K27me3, plays a role in maintaining imprinted silencing of some genes specifically in the placenta or its precursor tissue (Mager et al., 2003; Terranova et al., 2008). EHMT2, which deposits H3K9me2, may play a role specifically in EXEL imprinted silencing of genes in the placenta (Nagano et al., 2008; Wagschal et al., 2008). Additionally, early embryonic lethality of the *Eed* (PRC2) hypomorph been shown to be due to a defect in placenta development (Wang et al., 2002) indicating an essential role for H3K27me3 in placenta. Despite this, the H3K27me3 modification deposited by PRC2 is globally low in TS cells and XEN stem cells that represent the pre-implantation trophectoderm and primitive endoderm which generates the trophectoderm of the placenta and the ysE of the VYS (Rugg-Gunn et al., 2010). Given these reports we investigated expression of components of histone modifying complexes in ysE, fetal liver and cystic EBs by RNA-seq. We found that the PRC2 component *Ezh2* showed significantly higher expression in fetal liver and cystic EBs compared to ysE, while other PRC2 components *Suz12*, *Eed*, and *Ezh1* showed no significant difference between the tissues (Fig. 3F). Other components of histone modifying complexes *Rnf2*, *Mll1*, *Ash1*, and *Ehmt1* also showed no significant differences between the tissues. *Ehmt2* did show higher expression in E12.5 and E9.5 ysE than fetal liver and cystic EBs, but this difference was not statistically significant. Since significant expression differences between ysE and cystic EBs were seen for the histone modifying enzyme *Ezh2*, we examined total levels of the histone modifications H3K27me3, H3K4me3, H3K27ac, H3K9me2 and H3K9me3 in ysE, fetal liver and cystic EBs and compared them to undifferentiated ES cells and VYS by Western blotting (Fig. 3G). No difference in the total levels of these modifications was detected between these tissues. Although it has been shown that extra-embryonic stem cells in the pre-implantation embryo show a low level of H3K27me3 (Rugg-Gunn et al., 2010), our result is consistent with immunocytochemistry data indicating that post-implantation extra-embryonic lineages gain H3K27me3 (Rugg-Gunn et al., 2010), and indicates that cystic EBs represent a post-implantation tissue. As an additional marker of chromatin regulation, we examined expression of the enhancer associated CBP/p300 interacting protein CITED1, that is expressed in extra-embryonic VE and later in ysE (Dunwoodie et al., 1998), and is required for placental development (Rodriguez et al., 2004). Expression comparison by RNA-seq showed that *Cited1* expression in ysE was significantly higher than in cystic EBs and fetal liver (Fig. 3F). Taken together these results demonstrate that in addition to failing to display EXEL imprinted expression, cystic EBs do not possess epigenetic features of ysE such as DNA hypomethylation, elevated expression of IAPEz retroviral repeats, the *Tex19.1* genome defense gene, and the *Cited1* gene. Additionally, high expression levels of chromatin modifying enzyme *Ezh2* in cystic EBs more resemble the embryonic fetal liver, than the extra-embryonic ysE.

### The gene expression pattern of cystic EBs is distinct from the yolk sac endoderm

**Fig. 4:**
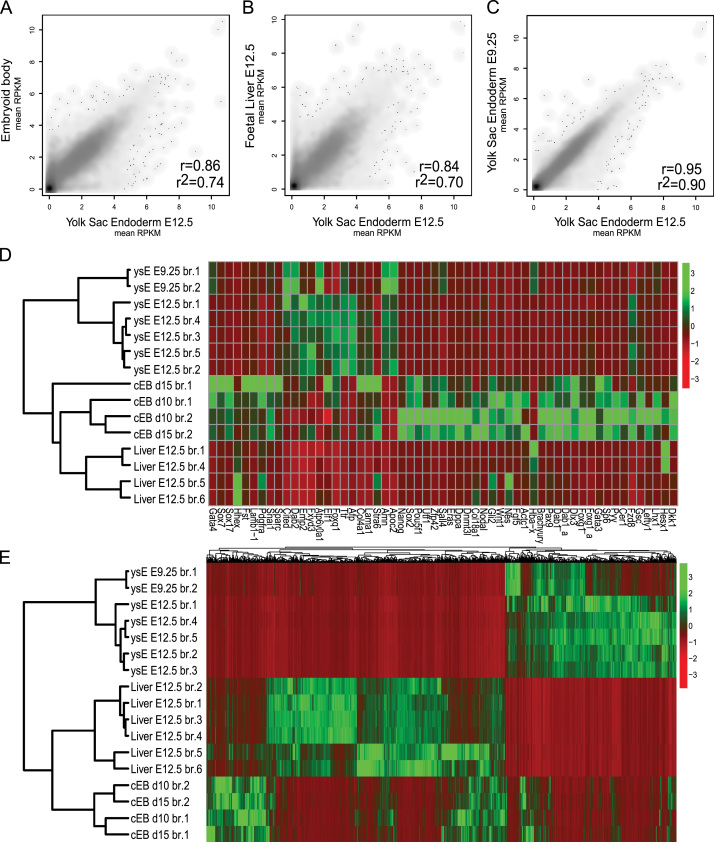
Transcriptome analysis shows that cystic EBs resemble fetal liver more than yolk sac endoderm. (A–C) Scatter plots comparing mean RPKM values from RNA-seq of yolk sac endoderm (ysE) and cystic EBs shows a weak correlation (left: *r*^2^=0.74), more similar to distantly related tissues such as E12.5 yolk sac endoderm and E12.5 fetal liver (middle: *r*^2^=0.70), than to closely related E12.5 and E9.25 yolk sac endoderm (right: *r*^2^=0.90). *r* is the Pearson coefficient of correlation. (D) Supervised clustering by expression of selected developmental markers shows a high similarity in expression patterns of ysE from two developmental stages (E12.5 and E9.25), while the expression pattern of cystic EBs clusters closer to the expression pattern of E12.5 liver. (E) Unsupervised classification by all RefSeq protein coding genes that show a highly significant (*P*<10^−3^) difference in expression between E12.5 fetal liver and E12.5 ysE (Fig. S4E), showed that cystic EBs cluster closer to fetal liver than to ysE. Hierarchical clustering for (D) and (E) detailed in Supplementary materials.

The expression of some gene markers, the imprinted expression pattern and epigenetic features indicate that cystic EBs are distinct from the yolk sac endoderm and may represent a developmental stage more similar to the embryonic definitive endoderm. Although RT-PCR of marker genes indicates that cystic EBs have a definitive endoderm identity (Fig. 1D), assigning tissue identity based on a limited set of markers is dependent on proper selection and could be misleading. Moreover, expression of some individual markers may be altered in culture. For example, *in vivo Afp* is expressed in the embryonic, but not the extra-embryonic VE, whereas in culture both express *Afp* (Dziadek, 1978). Therefore, confident and unbiased classification of tissue identity requires whole transcriptome analysis. To enable this we performed RNA-seq of cystic EBs at d10 and d15 of differentiation and compared them to E9.25 and E12.5 ysE and E12.5 fetal liver (Fig. 4). The mean expression level of biological replicates of cystic EBs from two independently derived B6/129 ES cells at the d10 and d15 time points showed a high correlation level of *r*=0.99, *r*^2^=0.98 (Fig. S4A) with no significantly differentially expressed genes (Fig. S4C). Therefore, we treated all 4 cystic EB datasets as biological replicates in comparisons to ysE and fetal liver samples. The comparison between cystic EBs and E12.5 ysE showed a correlation of *r*^2^=0.74 (Fig. 4A). This correlation resembles that seen between distantly related tissues, such as E12.5 ysE and fetal liver (*r*^2^=0.70) (Fig. 4B), rather than the correlation between two closely related tissues like E9.25 and E12.5 ysE (*r*^2^=0.90) (Fig. 4C). To classify the type of endoderm contained in cystic EBs, we performed a supervised hierarchical cluster analysis of RNA-seq data from cystic EBs, ysE and fetal liver using a set of markers reported to distinguish different types of endoderm and different stages of ES cell differentiation (Bielinska et al., 1999; Cho et al., 2012). Samples from each tissue type clustered together, with cystic EBs clustering closer to fetal liver than ysE, while E9.25 and E12.5 ysE clustered close to each other (Fig. 4D). Principal component analysis showed that most variation was explained by tissue differences and that batch effects only had a minor effect on the hierarchical clustering of samples (Fig. S4D). Similar to fetal liver, cystic EBs expressed markers of visceral endoderm (*Cited1*, *Dab2*, *Atp6v0a1*, *Elf1*, *Ttr*, *Afp* and *Apoc2*) to a lower level than observed in E9.25 and E12.5 ysE. Cystic EBs also expressed markers of embryonic VE and definitive endoderm (*Foxg1a*, *Gata3*, *Sp6*, *Pyy*, *Cer1*, *Gsc*, and *Lefty1*) (Fig. 4D), as was indicated by RT-PCR using a more limited panel of markers (Fig. 1D). Notably, cystic EBs also expressed genes associated with early endoderm development (*Gata6*, *Sox7* and *Sox17*), parietal endoderm (*Fst*, *Snai1*, *Pth1r*, *Pdgfra*, *Lamb1-1* and *Sparc*) and pluripotency-associated genes (*Nanog*, *Pou5f1*, *Sox2*, *Utf1*, *Zfp42*, *Eras*, *Dppa5*, *Dnmt3l*, *Col18a1*, *Nodal* and *Gli2*) (Fig. 4D). This may indicate that they represent an earlier stage of development than the ysE and fetal liver samples. The pluripotency associated genes *Oct4* and *Rex1* are strongly down regulated during cystic EB differentiation (Fig. S1G), but with our analysis it cannot be distinguished if the expression of pluripotency markers at this level is characteristic of cystic EBs or due to a sub-population of undifferentiated cells. To further classify the endoderm of EBs in an unbiased transcriptome-wide way, we performed hierarchical clustering using genes that most strongly distinguish E12.5 ysE and fetal liver (3133 genes showing *P*<10^−^^3^) (Fig. S4E). The resulting dendrogram showed a similar picture with samples clustering by tissue type, and cystic EBs clustering closer to fetal liver than ysE (Fig. 4E). In agreement with this, a direct comparison of expression patterns between cystic EBs and fetal liver showed a higher correlation (*r*^2^=0.79) than a direct comparison between cystic EBs and ysE (*r*^2^=0.74) (Figs. 4A and S4B). Together these results show that the expression profile of cystic EBs is more similar to fetal liver than ysE, indicating that the endoderm contained in cystic EBs resembles more embryonic definitive endoderm than extra-embryonic ysE.

### Cystic EBs have a different regulatory network than the yolk sac endoderm

**Fig. 5:**
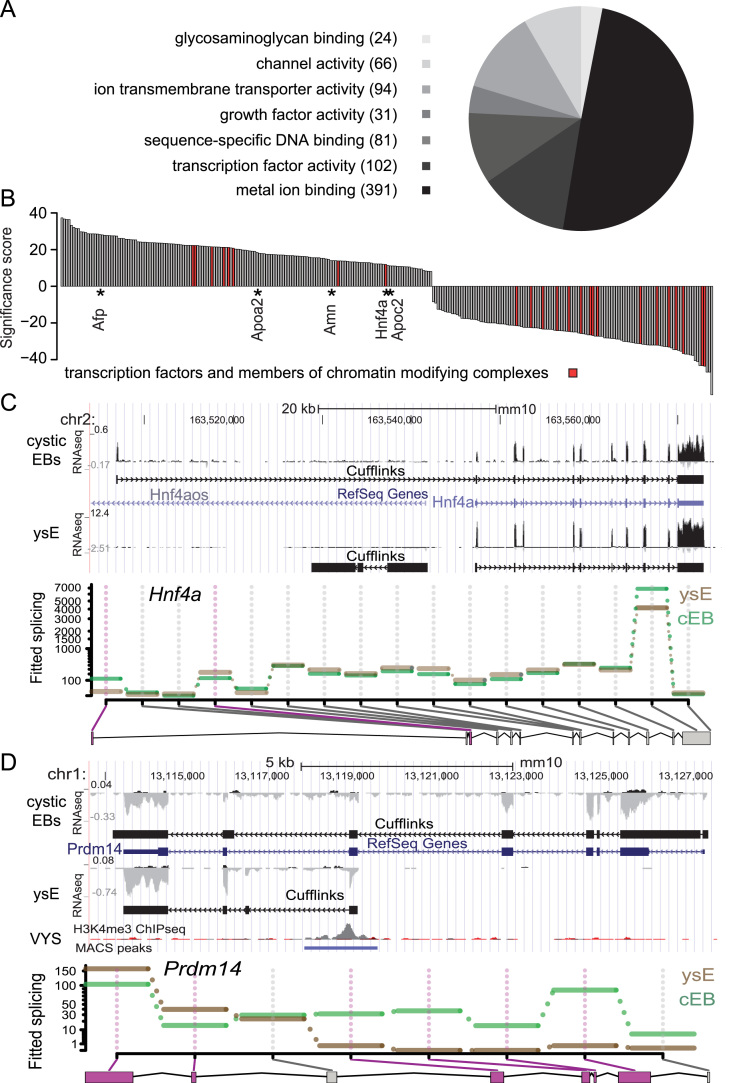
Cystic EBs have a different regulatory network than the yolk sac endoderm. (A) Pie chart representation of gene ontology (GO) analysis of differentially expressed genes between E12.5 ysE and cystic EBs. Significantly differentially expressed genes detected with a FDR of 1% were analyzed using DAVID (Huang et al., 2009). Over represented GO groups/terms (based on an EASE score <10^−3^) are listed. (B) Genes that show significant differential expression between both developmental stages of ysE (E9.25 and E12.5) and cystic EBs and E12.5 liver are plotted (272 genes, FDR<5%, Fig. S5E). To indicate the direction of differential expression, genes expressed higher in E12.5 ysE are displayed as positive (156 genes) and higher in cystic EBs as negative (118 genes) log_2_* P* values (Table S1). Members of chromatin modifying complexes and transcription factors are highlighted in red. Transcripts that were previously published as markers of ysE are marked with an asterisk. (C) E12.5 ysE expresses the RefSeq annotated shorter isoform of the endoderm specific regulator *Hnf4a*, while cystic EBs use an alternative promoter to express a longer form that includes a new first exon. Top: UCSC genome browser screenshot showing the RNA-seq tracks and *de novo* annotation using Cufflinks in cystic EBs and E12.5 ysE (black). Bottom: DEXSeq differential exon usage analysis shows significant differential first exon usage. (D) A novel isoform of the *Prdm14* transcription factor distinguishes cystic EBs and E12.5 ysE. Top: A UCSC genome browser screenshot showing RNA-seq for cystic EBs and E12.5 ysE, Cufflinks *de novo* annotation for both tissues (black), H3K4me3 ChIP-seq for E12.5 VYS (gray, input red) and significant H3K4me3 peaks called using the MACS program. The novel *Prdm14* isoform in ysE is supported by a H3K4me3 peak in VYS indicating a novel promoter for this gene. Bottom: DEXSeq differential exon usage analysis shows a significant differential isoform expression between E12.5 ysE and cystic EBs.

Analysis of the imprinted expression pattern, epigenetic profile and transcriptome of cystic EBs indicates that they contain endoderm that is distinct from the ysE. In order to identify the genes and pathways that are responsible for this difference we performed a differential expression analysis comparing cystic EBs with ysE. Using the DESeq BioConductor package we identified 3674 RefSeq transcripts differentially expressed between E12.5 ysE and cystic EBs (FDR<1%) (Fig. S5A). Gene ontology (GO) analysis of deregulated genes using DAVID software showed a significant (EASE index <10^−3^, *P*<10^−3^) overrepresentation of transcription factors (GO:0003700), sequence-specific DNA binding (GO:0043565) and growth factors (GO:0008083) indicating that the two tissues have a different regulatory network (Fig. 5A). To identify genes that are most likely to be essential for ysE development, and also genes expressed in cystic EBs that may be antagonistic to ysE development, we filtered for those genes that are significantly (FDR<5%) differentially expressed between both ysE stages and cystic EBs, and are also significantly different between E12.5 ysE and fetal liver (Fig. S5E). This identified 274 genes that distinguish ysE E9.5 and E12.5 from cystic EBs and fetal liver, 156 differentially expressed higher in ysE, and 118 differentially expressed higher in cystic EBs (Fig. 5B, Table S1). These 156 genes that distinguish ysE from cystic EBs included the known ysE markers *Afp*, *Apoa2*, *Amn*, *Hnf4a* and *Apoc2*. After this filtering step both the ysE and cystic EB gene sets still include a number of transcription factors and members of chromatin modifying complexes that may be involved in regulating tissue identity (Table S1).

Alternative splicing may result in isoforms that encode different protein products or that are differentially regulated by miRNAs, and has been suggested to affect differentiation and lineage commitment (Revil et al., 2010; Salomonis et al., 2010; Trapnell et al., 2010). Therefore, we compared ysE and cystic EBs to identify isoform differences that may contribute to their different cellular identity. A total of 8027 exons contained within 4192 Ensemble genes showed significant (FDR<5%) differential usage between ysE and cystic EBs (Fig. S5B). To further study the complexity of splicing in the different tissues and identify novel transcripts, we performed *de novo* transcriptome assembly using Cufflinks (Trapnell et al., 2010). To reduce artefacts we filtered for spliced transcripts with a minimum length of 500 bp leading to the identification of 13,288 loci (containing 45,638 transcripts) in E12.5 ysE, 14,360 loci (containing 52,555 transcripts) in cystic EBs and 13,878 loci (containing 55,903 transcripts) in fetal liver (Fig. S5C). We observed an 85% overlap between the putative transcription start sites (5'ends) of *de novo* annotated ysE transcripts and H3K4me3 ChIP-seq peaks from E12.5 VYS (containing ysE and VYS mesoderm) indicating that our *de novo* assembly approach was valid (Fig. S5D). The *de novo* assemblies included 479 loci in ysE, 289 loci in fetal liver and 216 loci in cystic EB that have not been previously annotated in publicly available gene or EST databases (Fig. S5C). From the putative novel ysE loci, 198 out of 479 were not identified in cystic EBs. Consistent with the DEXSeq analysis of Ensembl genes (Fig. S5B), many *de novo* assembled loci showed different isoforms usage between ysE and cystic EBs. This is illustrated by the example of two genes known to play a role in endoderm development. HNF4A is a transcription factor involved in regulating the transcriptional program in diverse endoderm tissues. It is significantly differentially expressed, nearly 20 fold higher in E12.5 ysE compared to cystic EBs, while there is no significant expression difference between cystic EBs and E12.5 liver (Fig. 5B, Table S1). Additionally, *de novo* assembly and differential exon usage analysis indicated that while the canonical *Hnf4a* isoform was present in ysE, cystic EBs had a longer form with an alternative exon 1 and TSS (Fig. 5C). Although this difference may or may not affect the protein sequence and molecular function of this important endoderm transcription factor, indicating that it is regulated differently and may thus also explain the significant difference in expression level between the tissues. PRDM14 is a transcription factor involved in early development and pluripotency that is expressed in primordial germ cells, the inner cell mass and ES cells and has been linked to the regulation of their unusual epigenetic state by DNA demethylation *via* the TET pathway (Okashita et al., 2014). Additionally, it has been shown to block mouse ES cells from differentiating down the extra-embryonic endoderm lineage *in vitro* (Salomonis et al., 2010). *Prdm14* was expressed in cystic EBs consistent with the role of PRDM14 as a suppressor of extra-embryonic endoderm differentiation, and our results indicating that cystic EBs contain embryonic, and not extra-embryonic endoderm, although we cannot exclude that at least part of this expression comes from remnant undifferentiated cells as indicated by expression of other pluripotency markers in cystic EBs (Fig. S1G). However, we were surprised when we also detected robust *Prdm14* expression in both E9.25 and E12.5 ysE. *De novo* assembly and differential exon usage analysis showed that while cystic EBs expressed the canonical form of *Prdm14*, an alternative promoter was used in ysE to express an isoform that included only the last four 3' exons. An H3K4me3 ChIP-seq peak from E12.5 VYS overlapped the 5' end of the predicted transcript confirming use of an alternative promoter (Fig. 5D). Possible translational start sites for all 3 open reading frames are present at the start or slightly upstream of the detected expression including a putative ~200 amino-acid sequence in frame with the known PRDM14 isoform. However, the ysE specific isoform would lack the N terminal and a part of the PR domain essential for the interactions with TET proteins (Okashita et al., 2014) indicating that if this isoform is biologically active its mode of action will differ from the canonical isoform. In summary, the large number of differentially expressed genes between ysE and cystic EBs are enriched for regulators of transcription, plus differential isoform usage between the tissues including important regulators of endoderm identity, indicating that cystic EBs have a regulatory network distinct from ysE.

## Discussion

The regulation of imprinted expression in extra-embryonic lineages appears to differ from embryonic lineages, but investigation of the mechanism is limited by the lack of a validated *in vitro* cell differentiation system that mimics the development of EXEL tissues. The differentiation of mouse ES cells into cystic EBs has been reported to form structures analogous to the VYS of a mid-gestation embryo that includes the ysE extra-embryonic lineage. Therefore, we investigated if cystic EBs could be used to model EXEL specific imprinted expression as seen in ysE (Hudson et al., 2011). We developed a protocol that allowed efficient differentiation of ES cells into cystic EBs that reproduced the reported morphology and marker expression. However, we report here that cystic EBs do not show EXEL specific imprinted expression but instead resemble embryonic lineages in their imprinted expression pattern. Furthermore, our WGBS DNA methylation and transcriptome analysis indicates that cystic EBs contain embryonic definitive endoderm rather than ysE, explaining the observed imprinted expression pattern.

### Cystic EBs provide a reliable model of ML imprinted expression

Differentiation of ES cells into different lineages provides valuable *in vitro* models of development that are amenable to experimental manipulation, enabling more rapid and cost effective testing of hypotheses than in animal models. Previously, ES differentiation into monolayers, standard EBs (hanging drop culture) and the neuronal lineage have been reported to recapitulate the developmental dynamics of imprinted expression for individual ML imprinted genes (Kohama et al., 2012; Latos et al., 2009; Meng et al., 2012; Wood et al., 2010). Here all tested ML genes showed imprinted expression in cystic EBs, demonstrating that differentiation into cystic EBs provides a robust model of ML imprinted expression that can be used in future studies.

### Cystic EBs show an embryonic rather than an extra-embryonic epigenetic state

Cystic EBs have been reported to contain a cell layer resembling the extra-embryonic ysE (Abe et al., 1996; Doetschman et al., 1985; Kurosawa, 2007; Yasuda et al., 2009). Therefore, it was expected that cystic EBs would show an epigenetic state similar to that seen in the extra-embryonic ysE. However, we found that cystic EBs do not show EXEL-specific imprinted expression, an epigenetic hallmark of extra-embryonic lineages (Hudson et al., 2011). Moreover, cystic EBs showed a relatively high level of DNA methylation, both globally and on specific genic and repeat elements, that was similar to embryonic liver, and distinct from extra-embryonic ysE, which showed relatively low DNA methylation levels. This shows that ysE is globally hypomethylated as shown for the extra-embryonic placenta (Popp et al., 2010). Related to this, in comparison to embryonic tissues and cystic EBs, the ysE showed high expression of IAPEz repeats as seen in placenta, and high expression of the genome defense gene *Tex19.1* that is known to suppress expression of LINEs expression in placenta where they are lowly methylated (Reichmann et al., 2013). Thus our data indicate that, contrary to expectation, cystic EBs have an embryonic-like rather than extra-embryonic epigenetic state. Although mouse ES cells are derived from E3.5 embryos prior to the differentiation of the primitive endoderm, they have been suggested to resemble a later stage of epiblast development where differentiation down the primitive endoderm lineage leading to the ysE may no longer be possible. In agreement with this ES cells injected into a blastocyst or morula contributed to the extra-embryonic lineages at a very low frequency (Beddington and Robertson, 1989) or did not contribute at all (Lallemand and Brulet, 1990). Contribution of ES cells to the extra-embryonic lineages may be improved by culturing in 2i media plus LIF and then selecting cells positive for the extra-embryonic endoderm marker *Hhex* for injection into blastocysts, although contribution to ysE was still extremely low (Morgani et al., 2013). Early embryos also show imprinted X-inactivation that is retained in extra-embryonic lineages, but lost in the epiblast that undergoes random X-inactivation. Consistent with an epiblast identity, female ES cells undergo random X-inactivation upon differentiation (Murakami et al., 2011). This has been confirmed in the endoderm layer of cystic EBs, showing that cystic EBs lack this epigenetic hallmark of extra-embryonic tissues (Sado et al., 1996). These reports together with our results indicate that cystic EBs have an embryonic epigenetic phenotype.

### Cystic EBs contain definitive endoderm rather than yolk sac endoderm

Cystic EBs have a VYS-like bilaminar morphology and were thought to contain ysE typical of E10–E13.5 (Abe et al., 1996; Doetschman et al., 1985), but our analysis of their epigenetic state indicates that they represent an embryonic tissue type. Gene ontology analysis, the large number of differentially expressed genes and differentially used splice variants confirmed that cystic EBs are a tissue distinct tissue from ysE. We showed by hierarchical clustering of both DNA methylation and gene expression patterns that cystic EBs cluster closer to the definitive endoderm tissue embryonic liver than the ysE. As a mixed tissue that contains other cell types in addition to endoderm, it was not expected that cystic EBs would be identical to either embryonic liver or ysE, but these global analyses do indicate the type of endoderm that they contain. The lack of *Sfmbt2* imprinted expression, which only shows imprinted expression in both embryonic and extra-embryonic lineages prior to E7.5, indicates that cystic EBs represent a later developmental stage. Most reported endoderm marker genes are expressed in more than one type of endoderm during development making it difficult to precisely classify the developmental stage of the endoderm contained in cystic EBs. However, cystic EBs do show expression of definitive endoderm markers such as *Lefty1* and *Pyy*, while the expression of *Cer1* and *Hesx1* and faint expression of *Otx1* indicates similarity with the developing foregut endoderm (Zorn and Wells, 2009). This is consistent with the formation of contracting cardiomyocytes in cystic EBs, which *in vivo* are induced by foregut endoderm and begin their pulsating action post E8.25, indirectly indicating the identity of the endoderm present in cystic EBs (Pal and Khanna, 2005; Zorn and Wells, 2009). These data all indicate that cystic EBs contain endoderm similar to embryonic endoderm around E8.5.

## Conclusions

Here we use analysis of imprinted expression, together with methylome and transcriptome analysis to show that extra-embryonic yolk sac endoderm (ysE) has an epigenetic state similar to that reported for extra-embryonic placenta. In contrast cystic EBs previously reported to contain ysE, display an embryonic epigenetic state and contain embryonic rather than extra-embryonic endoderm, thus they cannot be used as a model of extra-embryonic tissues as previously suggested (Abe et al., 1996; Doetschman et al., 1985; Kurosawa, 2007; Yasuda et al., 2009). However as we show here, EBs do offer a robust *in vitro* model for the analysis of ML imprinted expression, adding to previous reports that EB differentiation can model important developmental processes including cardiomyocyte development and haematopoiesis (Dang et al., 2002; Doetschman et al., 1985; Pal and Khanna, 2005). While our results do not exclude that EB differentiation may be directed down the primitive endoderm lineage leading to ysE, for example by overexpression of GATA factors (Fujikura et al., 2002), our RNAseq expression data shows that cystic EBs highly express factors associated with visceral endoderm specification such as *Gata4, Gata6, Bmp4* and *Hhex* (Artus et al., 2012; Fujikura et al., 2002; Morgani et al., 2013). Thus, either these factors are insufficient under the conditions used here or cystic EBs may expresses genes that are antagonistic to ysE development. The genomic data presented here from two developmental stages of ysE, which in contrast to placenta studies contains a homogenous EXEL cell population, together with fetal liver and cystic EBs, a widely used developmental model, provides a valuable resource to the epigenetic and developmental community for further investigations.
