# Supplementary material for: Imprinted expression in cystic embryoid bodies shows an embryonic and not an extra-embryonic pattern
Source: Dev Biol. 2015 Jun 15;402(2):291–305. doi: 10.1016/j.ydbio.2015.04.010 (PMC4454777; doi:10.1016/j.ydbio.2015.04.010)
Supplement: Supplementary file 1 — Supplementary material [file mmc1.pdf]

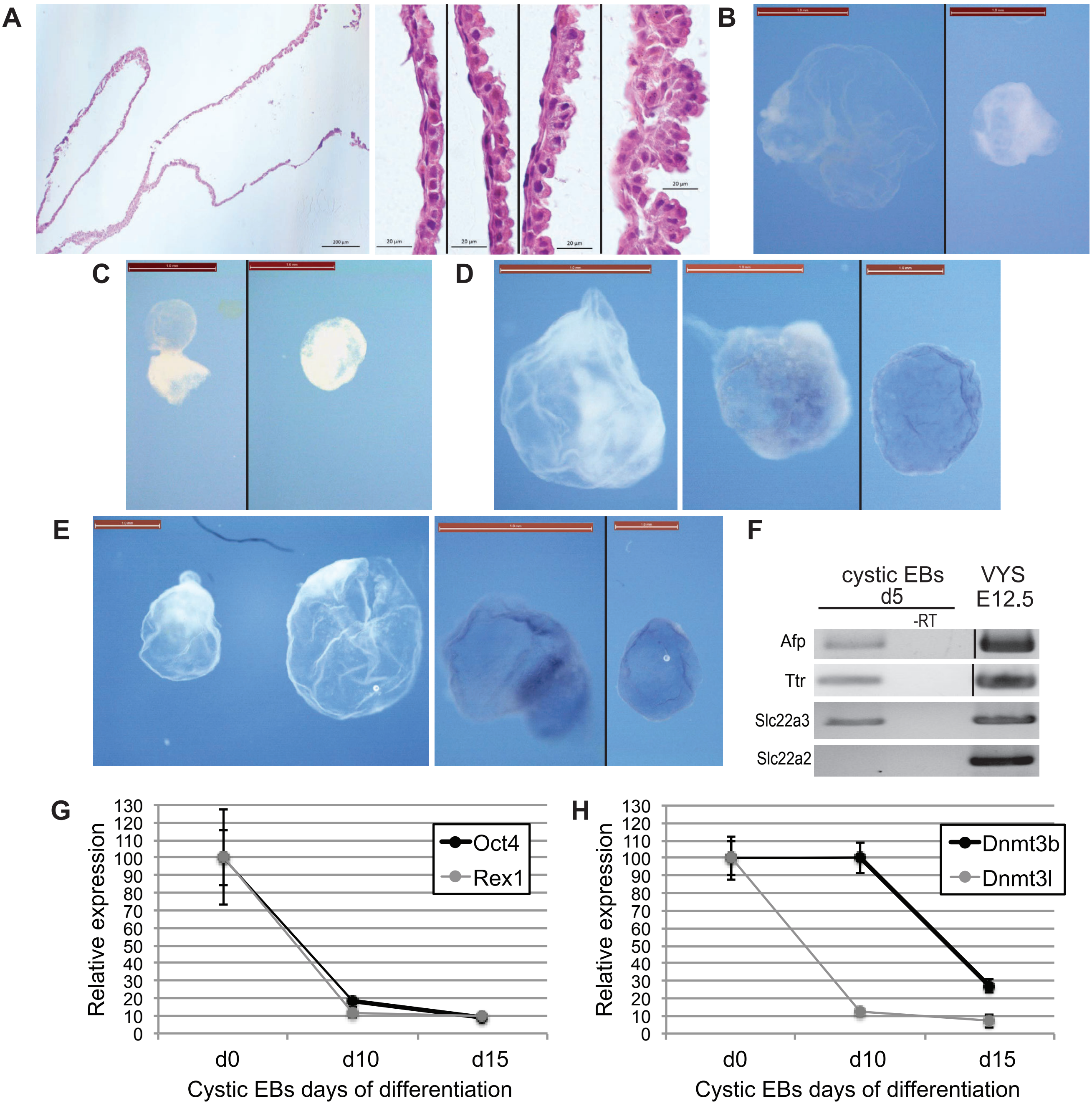

Figure. S1. The histology of visceral yolk sac (VYS) and detection of marker expression in cystic embryoid bodies (EBs) by in situ hybridization, RT-PCR and RT-qPCR. (A) Histological section of E12.5 VYS stained with Haematoxylin & Eosin showing a double layer organization with a columnar epithelium outer endoderm layer and inner mesoderm layer (scale bar left: 200μm, right: 20μm). No signal for an Afp sense probe (B) or a Ttr sense probe (C) was detected in cystic EBs by whole mount in situ hybridization (negative controls for Fig. 1G). Lefty1 (D) and Pyy (E) expression was detected in cystic EBs by whole mount in situ hybridization using antisense probes (right panels), whereas no signal was detected using sense probes (left panels). Scale bar for whole mount in situ hybidisations: 1mm. (F) EBs differentiated for 5 days with the cystic EB protocol showed expression of *Afp*, *Ttr*, and *Slc22a3* but not the specific yolk sac endoderm marker *Slc22a2* by RT-PCR, whereas E12.5 VYS showed expression of all markers. Primers for RT-PCR are given in Table S4. (G) Relative expression of the pluripotency markers *Oct4* and *Rex1* during cystic EB differentiation detected by RT-qPCR. (H) Relative expression of the *de novo* DNA methyltransferases *Dnmt3b* and *Dnmt3l* during cystic EB differentiation detected by RT-qPCR.. The cystic EB differentiation series assayed in G and H was differentiated from A9 p17 ES cells and expression levels were normalised to cyclophillin A. Error bars indicate the standard deviation of 3 technical replicates. Primers for RT-qPCR are detailed in Santoro et al 2013 Development 140, 1184-1195.

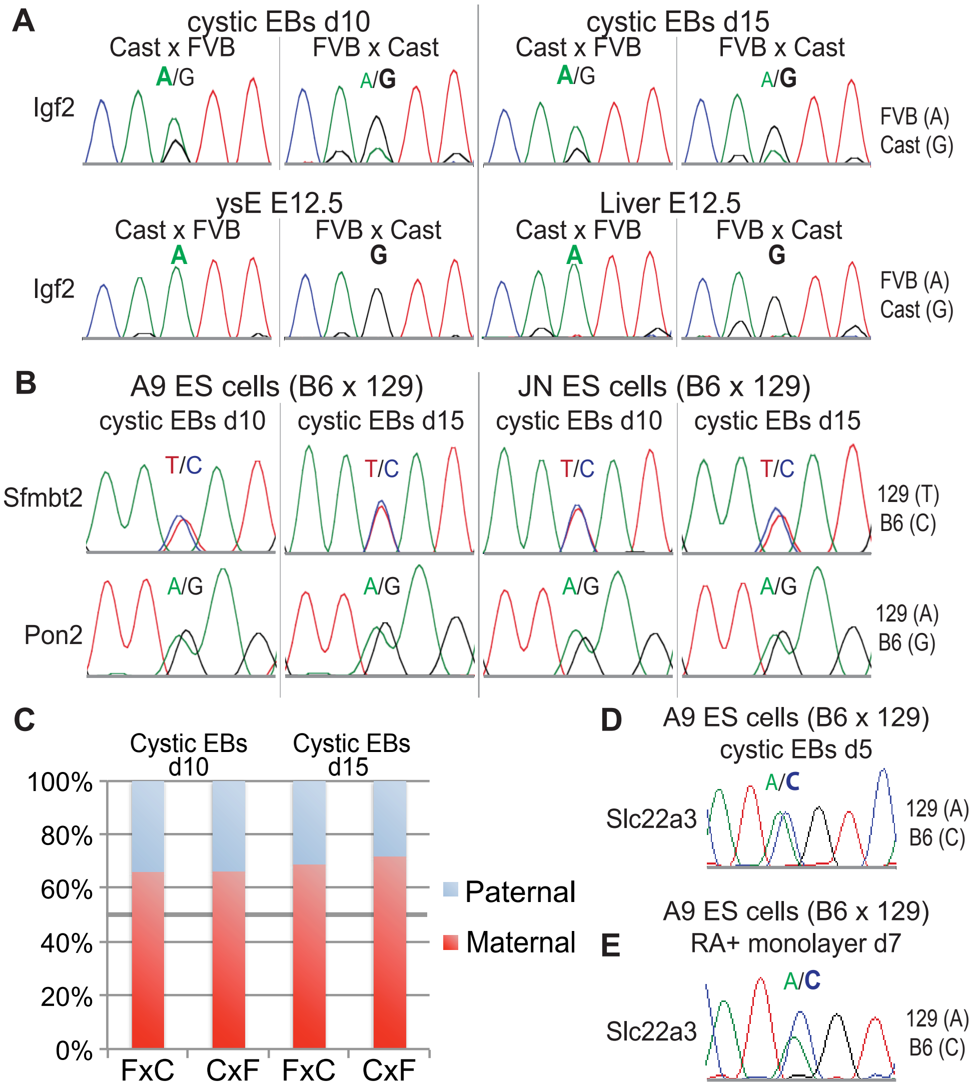

Figure S2. Extra-embryonic lineage (EXEL) specific imprinted gene expression in cystic embryoid bodies (EBs). (A) Sanger sequencing chromatograms of Igf2 showed biased reciprocal expression in cystic EBs differentiated for 10 and 15 days (top), while E12.5 ysE and liver showed full imprinted expression (bottom) using FVB and Cast reciprocal crosses. (B) Sanger sequencing chromatograms of Sfmbt2 and Pon2 showed biallelic expression in cystic EBs differentiated for 10 and 15 days (AW_A9 and JN cell lines with a F1 C57Bl/6 x 129SV background). (C) Quantification of the maternal bias observed in the sequencing tracks from Sanger sequencing chromatograms for Slc22a3 (Fig.2D top row) using the Mutation Surveyor® software. (D) Sanger sequencing chromatogram showing biallelic expression of *Slc22a3* at d5 of cystic EB differentiation. (E) Sanger sequencing chromatogram showing a moderate maternal biased expression of Slc22a3 in ES cells differentiated for 7 days as a monolayer with 0.27 µM retinoic acid, similar to the bias observed for cystic EBs (AW_A9 cell line with a F1 C57Bl/6 x 129SV background).

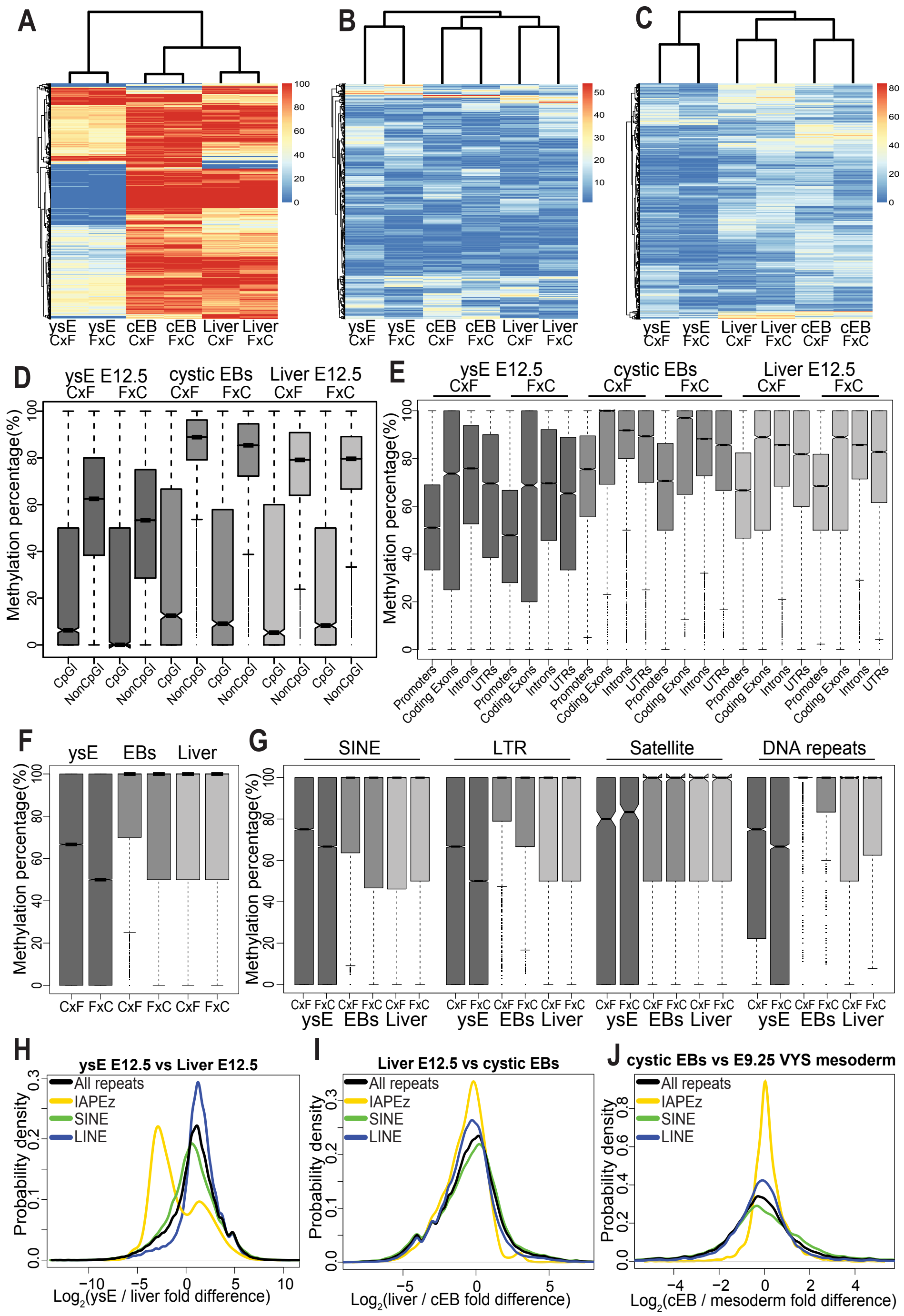

Figure S3. Cystic embryoid bodies (EBs) show epigenetic features more similar to fetal liver than yolk sac endoderm (ysE). (A) Hierarchical clustering of E12.5 ysE, cystic EBs and E12.5 liver by the methylation of all repeats (filtered as described in supplementary methods resulting in 1970 measurements). (B) Hierarchical clustering of E12.5 ysE, cystic EBs and E12.5 liver by the methylation of CpG islands (filtered as described in supplementary methods resulting in 972 measurements). (C) Hierarchical clustering of E12.5 ysE, cystic EBs and E12.5 liver by the methylation of promoters defined as 5kb regions centered around the transcriptional start site of RefSeq transcripts (filtered as described in supplementary methods resulting in 2568 measurements). (D) Boxplots indicating the methylation levels of CpG islands and the non-CpG island parts of the genome (analyzed in non-overlapping 5kb windows) in two replicates of E12.5 ysE, cystic EBs and E12.5 liver. (E) Boxplots indicating the methylation levels of genic structures (promoters, coding exons, introns and 5'+ 3' UTRs) in two replicates of E12.5 ysE, cystic EBs and E12.5 liver. (F) Boxplots indicating methylation levels of all repeats in two replicates of E12.5 ysE, cystic EBs and E12.5 liver. (G) Boxplot illustrating the methylation levels of SINE, LTR, Satellite and DNA repeat families in two replicates of E12.5 ysE, cystic EBs and E12.5 liver. (H-J) Kernel density plots showing the probability of repeats showing a fold difference expression in the two compared tissues (x-axis: log2 fold difference between two tissues, y-axis: probability density). (H) IAPEz repeats are highly expressed in E12.5 ysE compared to E12.5 liver, whereas other repeat classes show no significant difference in expression. (I) No repeat classes showed a significant difference in expression between E12.5 liver and cystic EBs. (J) No repeat classes showed a significant difference in expression between cystic EBs and E9.25 VYS mesoderm.

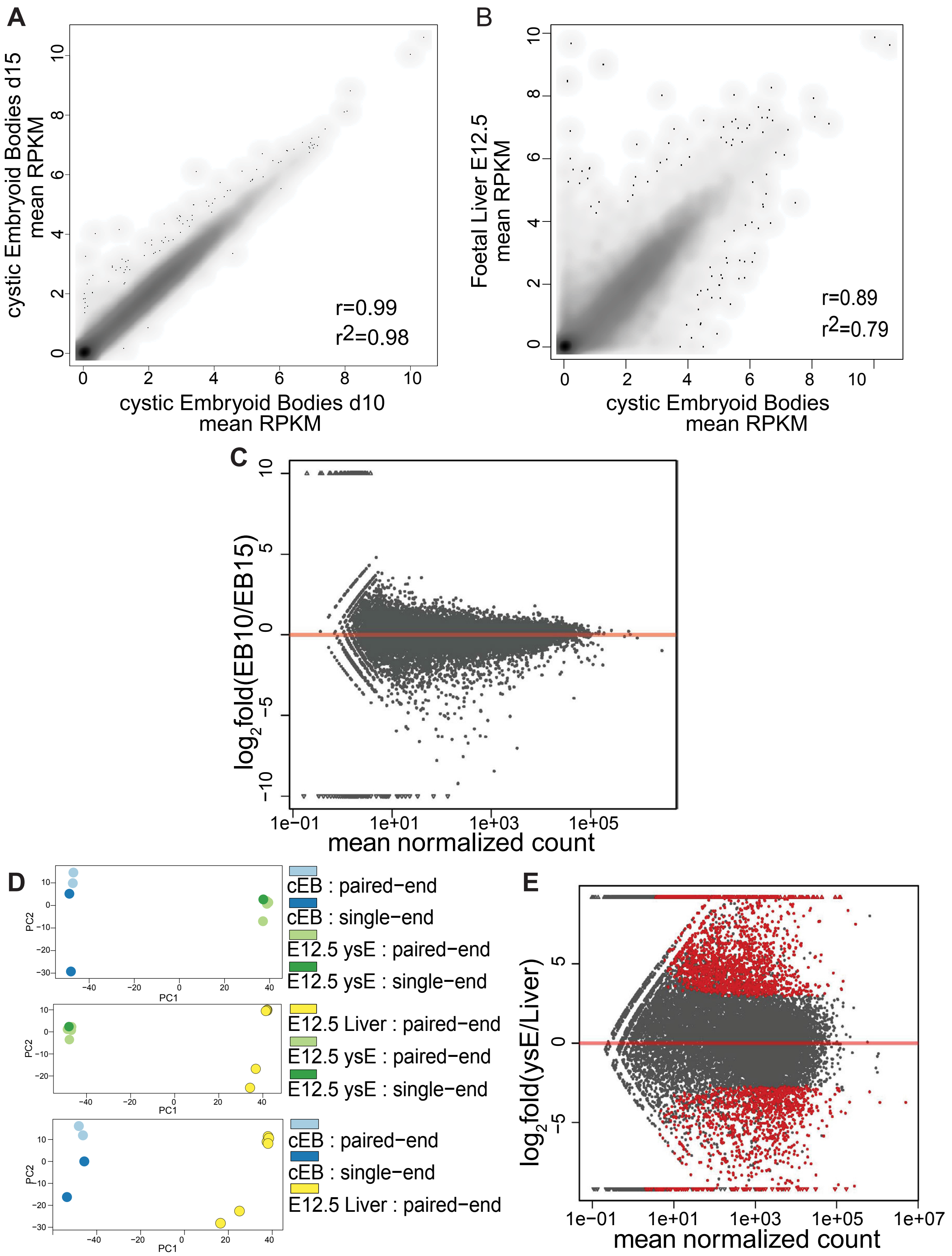

Figure S4. Differential gene expression analysis shows different stages of cystic embryoid body (EB) differentiation are similar whereas yolk sac endoderm and liver show many differences. (A) Cystic EBs differentiated for 10 days (d10, n=2) and 15 days (d15, n=2) show a strong correlation (r^2=0.98) in density representation scatter plots of mean RPKM values from RNA-seq. (B) In contrast, cystic EBs differentiated for 10 or 15 days (n=4) are less correlated with fetal liver (n=6) in density representation scatter plots of mean RPKM values from RNA-seq. (C) An MA plot ($\log_2$ fold change versus $\log_{10}$ mean expression) of differentially expressed transcripts identified between cystic EBs d10 and d15 shows no significant differences with a FDR cut-off of <0.05. A negative change represents the transcripts expressed higher in cystic EBs d15 and a positive change represents transcripts expressed higher in cystic EB d15. (D) A principal component analysis scatterplot shows that samples cluster according to tissue type with a minor batch affect according to RNA-seq technique. Data points are colour-coded according to the tissues type and shaded according to the sequencing technique. (E) An MA plot of differentially expressed transcripts identified in E12.5 ysE and liver. Genes showing a significant difference are coloured red (FDR <0.01). A negative change represents the transcripts expressed higher in cystic EBs and a positive change represents transcripts expressed higher in E12.5 ysE.

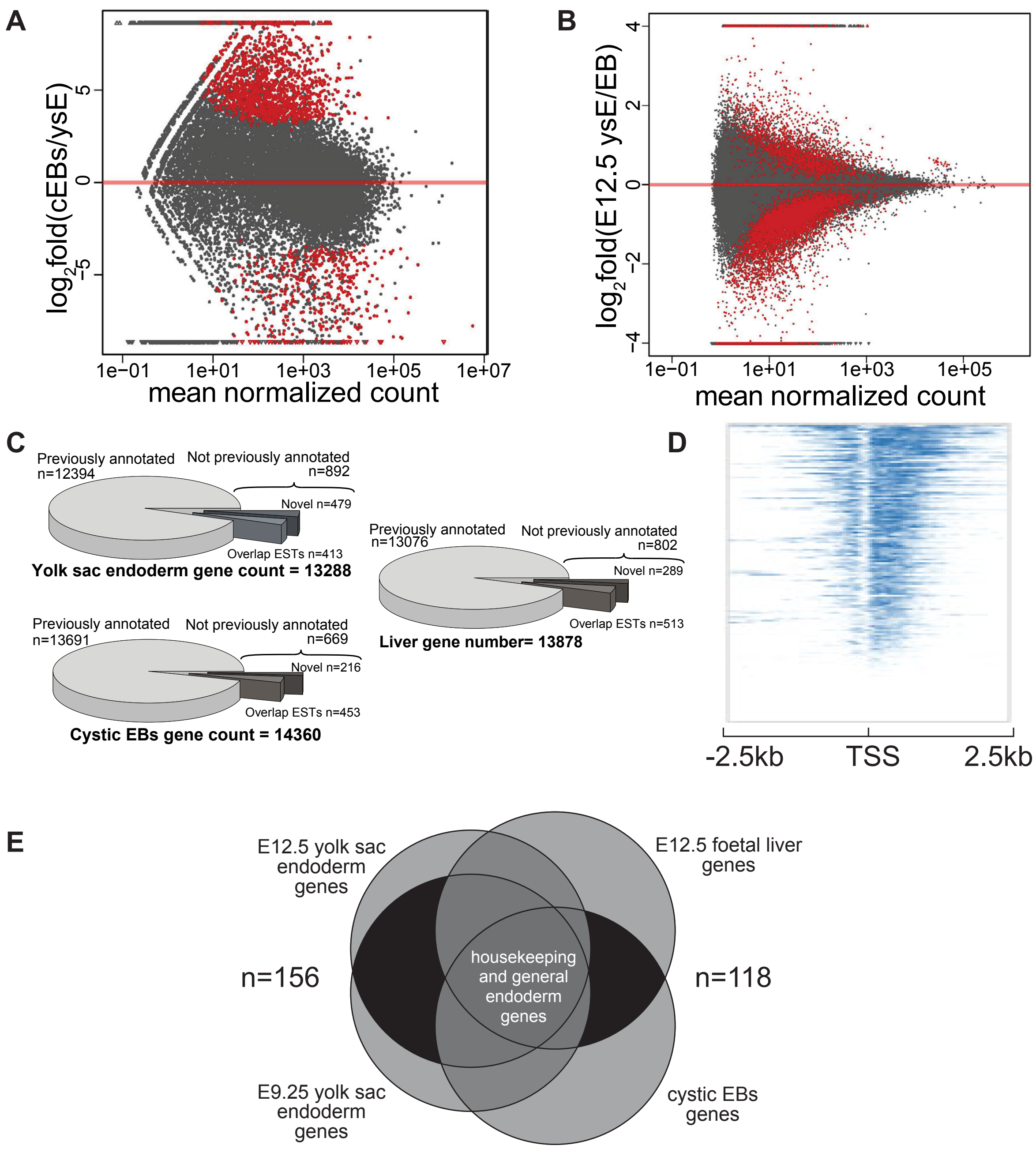

Figure S5. Transcriptome analysis of yolk sac endoderm (ysE) cystic embryoid bodies (EBs) and fetal liver. (A) An MA plot ($\log_2$ fold change versus $\log_{10}$ mean expression) of differentially expressed transcripts identified between E12.5 ysE and cystic EBs. A negative change represents the transcripts expressed higher in cystic EBs and a positive change represents the transcripts expressed higher in E12.5 ysE. Genes showing a significant difference are coloured red (FDR <0.01). (B) An MA plot ($\log_2$ fold change versus $\log_{10}$ mean expression) of differentially used exons identified between yolk sac endoderm and cystic EBs. A negative change indicates the exons used more in E12.5 ysE and a positive change represents the exons used more in cystic EBs. Exons showing a significant difference are coloured red (FDR <0.01). (C) Statistics of de novo gene locus annotations highlighting the putative novel genes identified in the sequenced tissues. (D) H3K4me3 enrichment validates de novo annotation of the ysE transcriptome. Heat map of H3K4me3 enrichment from E12.5 VYS ChIP-seq in a 5kb window centred over the predicted TSS from the de novo annotation from E12.5 ysE. (E) A Venn diagram illustrating the approach used in Fig. 5B to identify differentially expressed genes that distinguish ysE and cystic EBs (not to scale). Highlighted in black are the 156 genes expressed significantly higher in both E9.25 and E12.5 ysE compared to cystic EBs and fetal liver, and the 118 genes expressed higher in both cystic EBs and fetal liver compared to ysE. Values for E12.5 ysE and cystic EBs for these genes are given in Table S2.

Table S1. Differential expression between E12.5 ysE and cystic EBs for 272 genes that distinguish E9.5 and E12.5 ysE from cystic EBs and fetal liver.

Genes expressed differentially higher in E12.5 ysE than in cystic EBs (156 genes)						
RefSeq ID	ysE mean read counts	EBs mean read counts	fold change	P val	padj	gene name
NM_001111293	796.7	0.0	0.00E+00	5.81E-12	7.1E-09	Xlr5b
NM_053165	10743.3	12.6	1.17E-03	8.98E-12	8.7E-09	Clec2h
NM_001033879	2733.8	2.0	7.23E-04	1.12E-11	9.8E-09	Tubal3
NM_133977	2307265.1	1956.7	8.48E-04	1.09E-11	9.8E-09	Trf
NM_007994	27599.8	49.6	1.80E-03	9.71E-11	4.6E-08	Fbp2
NM_001161667	13171.2	9.8	7.46E-04	2.12E-10	8.0E-08	Acox2
NM_053115	12952.1	10.2	7.86E-04	3.16E-10	1.0E-07	Acox2
NM_025325	10050.1	15.9	1.58E-03	3.51E-10	1.1E-07	Haao
NM_133862	176282.0	539.7	3.06E-03	1.13E-09	2.6E-07	Fgg
NM_001165925	440.0	0.4	8.76E-04	1.23E-09	2.8E-07	Cldn14
NM_001165926	509.0	0.8	1.57E-03	1.50E-09	3.2E-07	Cldn14
NM_054094	8690.1	2.5	2.86E-04	2.32E-09	4.5E-07	Acsm1
NR_027641	5776.3	23.0	3.97E-03	2.42E-09	4.7E-07	Sec16b
NM_001081361	14030.6	36.8	2.62E-03	2.49E-09	4.8E-07	Mosc1
NM_033354	5776.7	23.4	4.05E-03	2.55E-09	4.9E-07	Sec16b
NM_001159986	5957.4	24.0	4.03E-03	2.64E-09	4.9E-07	Sec16b
NM_026152	11318.9	43.1	3.80E-03	3.25E-09	5.9E-07	Hoga1
NM_007423	9991957.7	46011.0	4.60E-03	3.64E-09	6.4E-07	Afp
NM_001033411	1036.7	2.4	2.32E-03	4.24E-09	7.1E-07	Gm826
NM_178669	9957.6	55.5	5.57E-03	4.58E-09	7.6E-07	Clrn3
NM_028078	4126.9	19.5	4.73E-03	4.64E-09	7.6E-07	Igsf5
NM_001177887	4146.4	20.3	4.91E-03	5.19E-09	8.2E-07	Igsf5
NM_027819	1023.7	3.4	3.34E-03	5.24E-09	8.2E-07	Ggt6
NM_001177886	2874.8	14.9	5.20E-03	5.72E-09	8.9E-07	Igsf5
NM_001127318	9453.8	49.3	5.21E-03	1.33E-08	1.8E-06	Gucy2c
NM_145067	9182.9	48.4	5.27E-03	1.34E-08	1.8E-06	Gucy2c
NM_027237	1094.2	5.4	4.91E-03	1.49E-08	2.0E-06	2010003K11Rik
NM_183094	358.1	1.6	4.55E-03	1.93E-08	2.3E-06	Xlr4c
NM_172778	53726.9	330.3	6.15E-03	1.97E-08	2.4E-06	Maob
NM_001045539	2567.0	22.6	8.82E-03	2.26E-08	2.6E-06	Xlr5a
NM_010231	66268.8	442.3	6.67E-03	2.38E-08	2.7E-06	Fmo1
NM_009034	4370.1	32.1	7.36E-03	2.46E-08	2.8E-06	Rbp2
NM_001033478	361.4	1.3	3.59E-03	5.03E-08	4.9E-06	Fam47e
NM_016674	14825.9	107.0	7.21E-03	5.32E-08	5.1E-06	Cldn1
NM_029269	121494.5	977.6	8.05E-03	5.74E-08	5.4E-06	Spp2
NM_010189	75629.6	501.8	6.63E-03	5.79E-08	5.4E-06	Fcgrt
NM_019500	299.9	0.9	3.13E-03	6.37E-08	5.8E-06	Cldn14
NM_001142706	70469.3	660.4	9.37E-03	6.96E-08	6.3E-06	Cfb
NM_008198	70639.6	664.3	9.40E-03	7.04E-08	6.3E-06	Cfb
NM_144512	7948.9	36.1	4.54E-03	7.22E-08	6.4E-06	Slc6a13

**NM_026656**	18180.1	133.2	7.32E-03	7.75E-08	6.8E-06	**Mcoln2**
**NM_001005846**	17459.7	129.4	7.41E-03	7.91E-08	6.9E-06	**Mcoln2**
**NM_001170571**	327.9	1.3	3.96E-03	8.10E-08	7.1E-06	**Fam47e**
**NM_146020**	3178.0	23.5	7.40E-03	8.72E-08	7.5E-06	**Gltpd2**
**NM_001081205**	8813.7	70.7	8.02E-03	9.08E-08	7.7E-06	**Nipal1**
**NM_144878**	7887.9	83.4	1.06E-02	1.04E-07	8.7E-06	**Fmo4**
**NM_001136237**	46758.3	380.0	8.13E-03	1.08E-07	9.0E-06	**Slc39a5**
**NM_028092**	46267.8	382.1	8.26E-03	1.13E-07	9.4E-06	**Slc39a5**
**NM_001110784**	11600.5	116.0	1.00E-02	1.26E-07	1.0E-05	**Xlr3a**
**NM_028051**	45038.9	387.3	8.60E-03	1.31E-07	1.0E-05	**Slc39a5**
**NM_001030291**	634.0	5.5	8.62E-03	1.54E-07	1.2E-05	**Enpp7**
**NM_021332**	7747.6	65.3	8.42E-03	1.75E-07	1.3E-05	**Glp1r**
**NR_045956**	644.5	6.6	1.02E-02	1.91E-07	1.4E-05	**4930405D11Rik**
**NM_001161621**	919.8	9.6	1.04E-02	1.91E-07	1.4E-05	**Abp1**
**NM_001161622**	919.3	9.6	1.04E-02	1.92E-07	1.4E-05	**Abp1**
**NM_013839**	9752.8	88.8	9.11E-03	1.93E-07	1.4E-05	**Nr1h3**
**NM_001177730**	9881.9	93.3	9.44E-03	2.01E-07	1.4E-05	**Nr1h3**
**NM_029638**	956.8	10.3	1.08E-02	2.02E-07	1.4E-05	**Abp1**
**NM_007468**	376310.0	2811.7	7.47E-03	2.44E-07	1.6E-05	**Apoa4**
**NM_001177467**	615.1	5.5	8.94E-03	2.51E-07	1.7E-05	**Gm7030**
**NM_001033364**	48864.7	513.4	1.05E-02	2.67E-07	1.7E-05	**Cdhr2**
**NM_133867**	11006.6	139.4	1.27E-02	3.02E-07	1.9E-05	**Eps8l3**
**NM_021365**	761.8	8.4	1.10E-02	3.19E-07	2.0E-05	**Xlr4b**
**NM_001163504**	4122.2	19.2	4.67E-03	3.29E-07	2.1E-05	**Nr1h4**
**NM_008290**	18720.2	282.1	1.51E-02	3.46E-07	2.1E-05	**Hsd17b2**
**NM_001161355**	21363.0	261.8	1.23E-02	3.51E-07	2.1E-05	**Timd2**
**NM_134249**	20916.9	258.8	1.24E-02	3.63E-07	2.2E-05	**Timd2**
**NM_009108**	4420.2	19.5	4.41E-03	3.91E-07	2.3E-05	**Nr1h4**
**NM_001161356**	20583.1	258.8	1.26E-02	3.90E-07	2.3E-05	**Timd2**
**NM_031493**	180.3	0.0	0.00E+00	4.04E-07	2.4E-05	**Xlr5c**
**NM_001163700**	4442.4	19.5	4.38E-03	4.14E-07	2.4E-05	**Nr1h4**
**NM_009692**	416208.1	5573.3	1.34E-02	5.02E-07	2.8E-05	**Apoa1**
**NM_145365**	16486.5	235.5	1.43E-02	5.66E-07	3.0E-05	**Creb3l3**
**NM_028104**	2322.3	29.0	1.25E-02	7.97E-07	3.9E-05	**Ppp1r14d**
**NM_011395**	5161.3	78.1	1.51E-02	8.09E-07	4.0E-05	**Slc22a3**
**NM_010197**	10717.8	159.4	1.49E-02	8.90E-07	4.3E-05	**Fgf1**
**NM_008670**	313.8	4.5	1.42E-02	1.12E-06	5.1E-05	**Naip1**
**NM_001195596**	1589.2	24.2	1.53E-02	1.15E-06	5.2E-05	**2010003K15Rik**
**NM_026779**	10300.4	136.5	1.32E-02	1.40E-06	6.1E-05	**Mocos**
**NM_023219**	1013.4	14.3	1.41E-02	1.59E-06	6.7E-05	**Slc5a4b**
**NM_175370**	446.2	6.7	1.50E-02	1.74E-06	7.2E-05	**Als2cr12**
**NM_013474**	99921.5	2074.8	2.08E-02	1.89E-06	7.8E-05	**Apoa2**
**NM_178257**	10438.0	195.6	1.87E-02	2.70E-06	1.0E-04	**Il22ra1**
**NM_174846**	12167.6	231.3	1.90E-02	3.74E-06	1.4E-04	**Glyctk**
**NM_001039586**	11603.1	229.0	1.97E-02	4.25E-06	1.5E-04	**Glyctk**

**NR_045443**	1258.5	34.2	2.72E-02	4.43E-06	1.6E-04	**1700019B21Rik**
**NM_011367**	97.1	1.7	1.79E-02	5.62E-06	1.9E-04	**Shbg**
**NR_015596**	1425.8	26.0	1.82E-02	5.68E-06	1.9E-04	**4930442L01Rik**
**NM_194333**	5972.1	133.4	2.23E-02	5.75E-06	1.9E-04	**Slc23a3**
**NR_045442**	1163.1	34.0	2.92E-02	6.08E-06	2.0E-04	**1700019B21Rik**
**NM_001173500**	2351.5	55.0	2.34E-02	6.74E-06	2.2E-04	**G630090E17Rik**
**NM_019432**	22626.1	500.2	2.21E-02	7.13E-06	2.3E-04	**Tmem37**
**NM_016675**	22634.4	672.1	2.97E-02	7.12E-06	2.3E-04	**Cldn2**
**NM_172825**	950.4	29.2	3.07E-02	7.37E-06	2.3E-04	**Gpr128**
**NM_023059**	17218.7	341.7	1.98E-02	8.18E-06	2.5E-04	**Sigirr**
**NM_001177872**	2203.9	53.5	2.43E-02	8.99E-06	2.7E-04	**Trim15**
**NM_001144993**	17566.1	415.3	2.36E-02	9.20E-06	2.8E-04	**2810459M11Rik**
**NM_001144992**	17569.6	415.3	2.36E-02	9.19E-06	2.8E-04	**2810459M11Rik**
**NM_025806**	19873.6	583.5	2.94E-02	1.04E-05	3.1E-04	**Plbd1**
**NM_001024134**	2270.3	56.9	2.51E-02	1.08E-05	3.2E-04	**Trim15**
**NM_001159725**	9664.2	296.5	3.07E-02	1.17E-05	3.4E-04	**Rab17**
**NM_018816**	75391.1	2018.3	2.68E-02	1.25E-05	3.6E-04	**Apom**
**NM_008998**	10844.8	333.6	3.08E-02	1.26E-05	3.6E-04	**Rab17**
**NM_134420**	7467.1	180.6	2.42E-02	1.43E-05	4.0E-04	**Slc26a6**
**NM_023530**	14481.4	450.4	3.11E-02	1.67E-05	4.5E-04	**Pla2g12b**
**NM_001161714**	13108.9	377.8	2.88E-02	2.00E-05	5.2E-04	**Tgm1**
**NM_001161715**	13149.8	381.4	2.90E-02	2.04E-05	5.3E-04	**Tgm1**
**NM_019984**	12779.9	372.9	2.92E-02	2.08E-05	5.4E-04	**Tgm1**
**NM_175512**	3208.6	124.8	3.89E-02	2.08E-05	5.4E-04	**Dhrs9**
**NM_178413**	22709.1	632.4	2.78E-02	2.55E-05	6.4E-04	**Thnsl2**
**NM_001033929**	22610.9	630.4	2.79E-02	2.57E-05	6.4E-04	**Thnsl2**
**NM_001081642**	628.5	23.9	3.80E-02	3.28E-05	7.8E-04	**Xlr4a**
**NM_133226**	5711.1	206.9	3.62E-02	3.76E-05	8.6E-04	**Pdzd3**
**NM_033603**	26291.7	823.1	3.13E-02	6.11E-05	1.3E-03	**Amn**
**NM_007980**	2982.8	136.2	4.57E-02	6.16E-05	1.3E-03	**Fabp2**
**NM_029360**	564.1	23.1	4.09E-02	6.27E-05	1.3E-03	**Tm4sf5**
**NM_172938**	497.0	15.5	3.12E-02	6.79E-05	1.4E-03	**Scml4**
**NM_001199996**	66401.4	3179.3	4.79E-02	6.83E-05	1.4E-03	**4933402E13Rik**
**NM_001122660**	28.3	0.3	1.18E-02	7.22E-05	1.5E-03	**Gm10639**
**NM_001199997**	67065.0	3258.5	4.86E-02	7.24E-05	1.5E-03	**4933402E13Rik**
**NM_020577**	15312.0	677.6	4.43E-02	7.98E-05	1.6E-03	**As3mt**
**NM_021291**	8551.6	375.8	4.39E-02	9.72E-05	1.8E-03	**Slc7a9**
**NM_008125**	10878.6	568.7	5.23E-02	9.80E-05	1.8E-03	**Gjb2**
**NM_001199016**	8929.9	399.8	4.48E-02	9.90E-05	1.9E-03	**Slc7a9**
**NM_001199015**	8980.8	411.8	4.59E-02	1.07E-04	2.0E-03	**Slc7a9**
**NM_001252553**	39712.2	1935.1	4.87E-02	1.23E-04	2.2E-03	**Folr1**
**NM_001252554**	39412.8	1932.1	4.90E-02	1.26E-04	2.2E-03	**Folr1**
**NM_001033410**	1016.6	44.5	4.38E-02	1.27E-04	2.3E-03	**Gm757**
**NM_008034**	40397.3	2010.6	4.98E-02	1.32E-04	2.3E-03	**Folr1**
**NM_001252552**	40401.9	2010.8	4.98E-02	1.32E-04	2.3E-03	**Folr1**

**NR_028061**	3481.1	214.6	6.16E-02	1.53E-04	2.6E-03	**Gm8615**
**NM_001025384**	176.7	6.0	3.40E-02	2.03E-04	3.3E-03	**DXBay18**
**NM_001025383**	175.5	6.0	3.42E-02	2.08E-04	3.3E-03	**Gm14685**
**NM_001193309**	92799.0	5482.0	5.91E-02	2.14E-04	3.4E-03	**Morc4**
**NM_029413**	93614.4	5550.6	5.93E-02	2.17E-04	3.4E-03	**Morc4**
**NM_001113385**	5761.0	2.9	5.02E-04	2.42E-04	3.7E-03	**BC021785**
**NM_008261**	42486.2	2510.2	5.91E-02	2.65E-04	4.0E-03	**Hnf4a**
**NM_009695**	35258.2	2733.0	7.75E-02	3.81E-04	5.3E-03	**Apoc2**
**NM_021517**	43510.2	711.7	1.64E-02	4.54E-04	6.1E-03	**Pdzk1**
**NM_029494**	5723.1	390.3	6.82E-02	4.57E-04	6.1E-03	**Rab30**
**NM_001201390**	108.3	7.1	6.60E-02	4.73E-04	6.3E-03	**E330021D16Rik**
**NM_008221**	20344.1	1209.8	5.95E-02	5.43E-04	7.1E-03	**Hbb-y**
**NM_026680**	1908.0	129.6	6.79E-02	5.52E-04	7.1E-03	**Golt1a**
**NM_001146001**	42297.2	697.9	1.65E-02	5.77E-04	7.4E-03	**Pdzk1**
**NM_025572**	7495.7	467.7	6.24E-02	6.23E-04	7.8E-03	**2610528J11Rik**
**NM_001033423**	688.1	0.7	1.03E-03	6.38E-04	8.0E-03	**Gm773**
**NM_001081084**	239835.8	19847.4	8.28E-02	6.68E-04	8.3E-03	**Cubn**
**NM_001001489**	3911.3	2.9	7.40E-04	9.47E-04	1.1E-02	**BC021785**
**NM_001033185**	259.9	17.7	6.81E-02	1.03E-03	1.2E-02	**1700084C01Rik**
**NM_011683**	29.3	0.0	Inf	1.27E-03	1.4E-02	**Vmn1r51**
**NM_001025384_2**	166.8	10.8	6.50E-02	1.42E-03	1.5E-02	**DXBay18**
**NM_001025383_2**	166.0	10.8	6.53E-02	1.45E-03	1.5E-02	**Gm14685**
**NM_001113388**	2387.8	40.3	1.69E-02	2.54E-03	2.4E-02	**BC021785**
**NR_015519**	201.4	15.4	7.63E-02	3.14E-03	2.8E-02	**AI662270**
**NM_001085529**	166.6	8.8	5.26E-02	3.41E-03	3.0E-02	**Slc2a7**
**NR_033324**	12008.0	1521.5	1.27E-01	3.73E-03	3.2E-02	**BC065397**
**Genes expressed differentially higher in cystic EBs than in E12.5 ysE (118 genes)**						
**gene name**	**ysE mean read counts**	**EBs mean read counts**	**fold change**	**P val**	**padj**	**gene name**
**NM_080728**	0.7	7993.8	1.13E+04	1.53E-18	4.3E-14	**Myh7**
**NM_008440**	7.9	7454.2	9.39E+02	8.15E-15	3.3E-11	**Kif1a**
**NM_001110315**	7.9	7453.5	9.39E+02	8.16E-15	3.3E-11	**Kif1a**
**NM_021459**	0.0	932.7	Inf	9.10E-14	2.0E-10	**Isl1**
**NM_011443**	10.0	6823.1	6.85E+02	7.62E-14	2.0E-10	**Sox2**
**NM_133197**	1.1	987.8	9.26E+02	9.52E-14	2.0E-10	**Mcf2**
**NM_010910**	1.7	1601.0	9.64E+02	4.30E-13	8.0E-10	**Nefl**
**NM_025274**	9.9	4917.1	4.99E+02	6.23E-13	1.1E-09	**Dppa5a**
**NM_001080814**	5.2	2425.5	4.63E+02	4.30E-12	6.0E-09	**Fat3**
**NM_022879**	0.9	2877.9	3.26E+03	5.30E-12	7.1E-09	**Myl7**
**NR_033497**	0.0	239.8	Inf	7.10E-12	7.8E-09	**D830015G02Rik**
**NM_172399**	0.0	311.8	Inf	7.22E-12	7.8E-09	**A930038C07Rik**
**NM_007430**	0.0	576.6	Inf	8.98E-12	8.7E-09	**Nr0b1**
**NM_001081281**	0.0	202.8	Inf	2.21E-11	1.7E-08	**Trim55**
**NM_011889**	1.8	808.3	4.42E+02	3.06E-11	2.2E-08	**Sep-03**
**NM_010112**	4.2	1290.7	3.08E+02	4.00E-11	2.3E-08	**Efs**

**NM_008671**	0.5	350.1	6.56E+02	3.87E-11	2.3E-08	**Nap1l2**
**NM_023396**	1.5	577.5	3.80E+02	7.68E-11	3.9E-08	**Rprm**
**NM_010703**	0.7	385.4	5.43E+02	1.05E-10	4.7E-08	**Lef1**
**NM_001198565**	48.5	9107.2	1.88E+02	1.21E-10	5.3E-08	**Sulf1**
**NM_001198566**	48.2	8910.6	1.85E+02	1.34E-10	5.5E-08	**Sulf1**
**NM_172294**	47.9	8545.0	1.78E+02	1.57E-10	6.3E-08	**Sulf1**
**NM_009994**	3.2	800.3	2.52E+02	1.99E-10	7.6E-08	**Cyp1b1**
**NM_009189**	0.7	333.1	4.70E+02	2.14E-10	8.0E-08	**Six1**
**NM_028351**	3.7	890.3	2.42E+02	2.20E-10	8.0E-08	**Rspo3**
**NM_178394**	1.1	415.9	3.92E+02	2.21E-10	8.0E-08	**Jakmip1**
**NM_172430**	2.1	613.2	2.85E+02	2.25E-10	8.1E-08	**Sphkap**
**NR_002874**	0.0	521.5	Inf	2.37E-10	8.3E-08	**Has2as**
**NM_001113181**	0.9	289.0	3.36E+02	2.79E-10	9.4E-08	**Gria4**
**NM_009865**	0.0	156.9	Inf	2.97E-10	9.9E-08	**Cdh10**
**NM_011382**	14.3	2394.2	1.67E+02	3.77E-10	1.2E-07	**Six4**
**NM_009911**	5.3	1119.0	2.11E+02	4.03E-10	1.2E-07	**Cxcr4**
**NM_145123**	0.0	145.1	Inf	4.45E-10	1.3E-07	**Crtac1**
**NM_001077354**	4.3	851.5	1.96E+02	5.50E-10	1.6E-07	**C77370**
**NM_028882**	5.0	858.0	1.71E+02	6.30E-10	1.7E-07	**Sema3d**
**NM_018741**	0.0	135.4	Inf	7.33E-10	1.9E-07	**Igfbpl1**
**NM_025285**	3.5	1680.4	4.77E+02	8.27E-10	2.0E-07	**Stmn2**
**NM_008108**	1.9	372.6	1.95E+02	8.82E-10	2.2E-07	**Gdf3**
**NM_183171**	2.4	503.7	2.12E+02	1.44E-09	3.2E-07	**Fez1**
**NM_019691**	2.0	372.6	1.82E+02	2.10E-09	4.3E-07	**Gria4**
**NM_001113180**	2.0	370.7	1.81E+02	2.16E-09	4.3E-07	**Gria4**
**NM_198865**	0.5	179.3	3.49E+02	2.59E-09	4.9E-07	**Slitrk5**
**NM_007603**	44.0	5718.2	1.30E+02	2.69E-09	5.0E-07	**Capn6**
**NM_001204371**	0.0	111.3	Inf	3.03E-09	5.6E-07	**Oprk1**
**NM_011011**	0.0	110.3	Inf	3.21E-09	5.8E-07	**Oprk1**
**NM_008800**	6.1	762.7	1.25E+02	3.31E-09	5.9E-07	**Pde1b**
**NM_019784**	0.0	103.2	Inf	6.09E-09	9.2E-07	**Tex21**
**NM_028472**	9.6	1232.6	1.29E+02	6.18E-09	9.3E-07	**Bmper**
**NM_030708**	11.4	1384.5	1.22E+02	6.35E-09	9.5E-07	**Zfhx4**
**NM_001159532**	0.0	102.2	Inf	6.49E-09	9.7E-07	**Tex21**
**NM_007960**	4.3	501.5	1.17E+02	8.23E-09	1.2E-06	**Etv1**
**NM_001163154**	3.4	383.3	1.14E+02	1.03E-08	1.4E-06	**Etv1**
**NM_008741**	1.9	298.2	1.56E+02	1.07E-08	1.5E-06	**Nsg2**
**NM_001164504**	2.2	319.8	1.49E+02	1.40E-08	1.8E-06	**Rnf165**
**NM_172706**	2.1	172.5	8.08E+01	1.60E-08	2.1E-06	**9330182L06Rik**
**NM_009234**	159.9	13493.0	8.44E+01	1.79E-08	2.2E-06	**Sox11**
**NM_175432**	22.5	2106.4	9.36E+01	2.13E-08	2.5E-06	**Tmem132c**
**NM_023872**	4.6	561.7	1.21E+02	3.07E-08	3.3E-06	**Kcnq5**
**NM_001160139**	4.6	561.7	1.21E+02	3.07E-08	3.3E-06	**Kcnq5**
**NM_053144**	1.2	179.0	1.52E+02	3.29E-08	3.5E-06	**Pcdhb19**
**NM_010425**	1.9	171.1	8.81E+01	3.78E-08	3.9E-06	**Foxd3**

**NM_001081009**	8.3	712.3	8.63E+01	4.07E-08	4.1E-06	**Parp8**
**NM_183177**	3.4	331.9	9.64E+01	4.55E-08	4.5E-06	**Zfp811**
**NM_025681**	3.0	318.8	1.05E+02	4.88E-08	4.8E-06	**Lix1**
**NR_028107**	0.0	67.0	Inf	5.17E-08	5.0E-06	**1700058G18Rik**
**NM_010059**	4.9	454.9	9.30E+01	5.17E-08	5.0E-06	**Dmc1**
**NM_021452**	1.6	148.3	9.04E+01	5.99E-08	5.6E-06	**Kcnmb4**
**NR_015595**	0.3	98.4	3.35E+02	8.25E-08	7.2E-06	**2410137F16Rik**
**NM_133189**	1.6	107.2	6.70E+01	8.71E-08	7.5E-06	**Cacng7**
**NM_182809**	0.0	50.2	Inf	9.03E-08	7.7E-06	**Ntrk3**
**NM_053146**	0.5	109.0	2.31E+02	9.15E-08	7.8E-06	**Pcdhb21**
**NM_013904**	7.3	603.8	8.23E+01	9.39E-08	7.9E-06	**Hey2**
**NM_009152**	14.5	1043.2	7.19E+01	1.10E-07	9.2E-06	**Sema3a**
**NM_001159648**	1.7	155.6	9.29E+01	1.41E-07	1.1E-05	**Cntn1**
**NM_001243073**	14.5	991.7	6.83E+01	1.49E-07	1.2E-05	**Sema3a**
**NM_001243072**	14.5	990.9	6.83E+01	1.50E-07	1.2E-05	**Sema3a**
**NM_053242**	4.6	341.4	7.39E+01	1.62E-07	1.2E-05	**Foxp2**
**NM_007727**	1.7	149.1	8.90E+01	1.74E-07	1.3E-05	**Cntn1**
**NM_026380**	0.0	49.6	Inf	1.76E-07	1.3E-05	**Rgs8**
**NM_001159647**	1.7	148.0	8.84E+01	1.80E-07	1.3E-05	**Cntn1**
**NM_175549**	37.6	2425.6	6.44E+01	2.35E-07	1.6E-05	**Robo2**
**NM_010259**	5.2	355.6	6.80E+01	3.09E-07	2.0E-05	**Gbp1**
**NM_212435**	4.6	299.6	6.49E+01	3.32E-07	2.1E-05	**Foxp2**
**NM_010298**	5.2	301.0	5.81E+01	3.79E-07	2.2E-05	**Glrb**
**NM_008746**	0.0	38.5	Inf	4.16E-07	2.4E-05	**Ntrk3**
**NM_011824**	0.3	75.6	2.63E+02	4.22E-07	2.4E-05	**Grem1**
**NM_053142**	2.6	203.1	7.87E+01	4.75E-07	2.7E-05	**Pcdhb17**
**NM_001163691**	16.1	712.2	4.43E+01	6.28E-07	3.2E-05	**Cacna1h**
**NM_001033408**	5.8	327.4	5.62E+01	6.63E-07	3.3E-05	**Rbm44**
**NM_021415**	16.3	716.0	4.39E+01	6.66E-07	3.3E-05	**Cacna1h**
**NM_009182**	1.9	123.9	6.42E+01	7.32E-07	3.6E-05	**St8sia3**
**NM_001160371**	0.6	295.6	5.03E+02	9.57E-07	4.5E-05	**A830018L16Rik**
**NM_016663**	0.0	29.4	Inf	9.50E-07	4.5E-05	**Syt3**
**NM_001160370**	0.6	306.4	5.21E+02	9.74E-07	4.6E-05	**A830018L16Rik**
**NM_001114116**	0.0	29.3	Inf	9.80E-07	4.6E-05	**Syt3**
**NR_045053**	10.6	480.0	4.53E+01	1.12E-06	5.1E-05	**2610307P16Rik**
**NM_009062**	7.3	306.8	4.18E+01	1.39E-06	6.1E-05	**Rgs4**
**NR_045054**	12.0	499.5	4.16E+01	1.46E-06	6.3E-05	**2610307P16Rik**
**NM_018800**	0.6	57.1	9.71E+01	2.54E-06	9.9E-05	**Syt6**
**NM_007664**	84.2	2930.5	3.48E+01	3.23E-06	1.2E-04	**Cdh2**
**NM_001190352**	1.0	100.5	9.60E+01	3.78E-06	1.4E-04	**Tmem132b**
**NM_001081388**	0.5	53.7	1.05E+02	3.78E-06	1.4E-04	**Rimbp2**
**NM_198863**	2.9	125.5	4.26E+01	4.60E-06	1.6E-04	**Slitrk2**
**NM_001161431**	2.9	123.4	4.19E+01	4.90E-06	1.7E-04	**Slitrk2**
**NM_010861**	1.1	245.5	2.14E+02	5.12E-06	1.8E-04	**Myl2**
**NR_015455**	1.1	65.0	6.05E+01	5.51E-06	1.9E-04	**BC064078**

**NM_177615**	11.2	343.5	3.06E+01	1.32E-05	3.8E-04	**Slc26a10**
**NM_001085521**	2.1	77.1	3.63E+01	2.13E-05	5.5E-04	**Tmem90b**
**NM_013540**	6.7	198.3	2.97E+01	2.96E-05	7.2E-04	**Gria2**
**NM_001083806**	6.7	196.9	2.95E+01	3.04E-05	7.4E-04	**Gria2**
**NM_001160369**	4.5	202.8	4.55E+01	5.22E-05	1.1E-03	**A830018L16Rik**
**NM_177173**	4.5	202.5	4.55E+01	5.49E-05	1.2E-03	**A830018L16Rik**
**NM_001039195**	6.2	155.5	2.52E+01	6.72E-05	1.4E-03	**Gria2**
**NM_009144**	22.3	390.2	1.75E+01	9.96E-05	1.9E-03	**Sfrp2**
**NM_007831**	1.9	48.4	2.60E+01	1.48E-04	2.5E-03	**Dcc**
**NM_175506**	15.2	261.4	1.72E+01	1.72E-04	2.9E-03	**Adamts19**
**NM_010140**	21.4	241.2	1.13E+01	1.76E-03	1.8E-02	**Epha3**
**NM_010816**	72.7	607.2	8.36E+00	2.92E-03	2.6E-02	**Morc1**

Genes showing no significant differential expression between E9.25 and E12.5 ysE and no significant differential expression between cystic EBs and E12.5 fetal liver, that are significantly differentially expressed between ysE and cystic EBs/fetal liver (Fig. 5B, Fig. S5E, 272 genes, 156 genes higher in ysE, 118 genes higher in cystic EBs/fetal liver). Values for E12.5 ysE vs. cystic EBs are displayed calculated using the DESeq package from BioConductor.

Key: P val = *p* value, padj = *p* value adjusted with the Benjamini-Hochberg procedure, Inf = infinite fold change.

Table S2. ES cell media for deriving new ES cell lines

SR-ES Medium

DMEM (Gibco, cat.nr: 12430)	500ml
KO-Serum (Gibco, cat.nr: 10828)	100ml
Non-essential amino acids (100x)	6ml
Penicillin/Streptomycin (100x)	6ml
Natrium-Pyruvat (100x)	6ml
L-Glutamine (100x)	6ml
β-Mercaptoethanol (55mmol)	1092μl
LIF (Millipore, cat.nr: ESG1107)	120μl

ES Medium

DMEM (Gibco, cat.nr: 12430)	500ml
FCS (Gibco, cat.nr: 10270-106)	100ml
Non-essential amino acids (100x)	6ml
Penicillin/Streptomycin (100x)	6ml
Natrium-Pyruvat (100x)	6ml
L-Glutamine (100x)	6ml
β-Mercaptoethanol (55mmol)	1092μl
LIF (Millipore, cat.nr: ESG1107)	120μl

Table S3. Primer sequences

Primers for RT-PCR reactions (Fig. 1D-F) and whole mount *in situ* probes (*, Fig. 1G-H, Fig. S1)		
**Gene**	**Forward**	**Reverse**
Actb	CCACCATGTACCCAGGCATT	AGGGTGTAAAACGCAGCTCA
Afp*	AGAGAATGGCGTCAAACCTG	GGCTTTCCTCGTGTAACCAA
Amn	CAGGAGGCGACTCGGACCCA	CACTGCCGAGACGCTGCGAA
Gata4	CAGCCCAGTCCTGCACAGCC	GGGCCGGTTGATGCCGTTCA
Hnf4a	GGAGGAGGCAGGAGGAGGGC	ATGGCGCACAGGGCACTGAC
Ins2	CCATCAGCAAGCAGGAAGGT	GCTGGTGCAGCACTGATCTA
Lefty1*	GACCCTTTCAGGACACCTCAG	TGTGTGGGGACAGCCTCTTT
Mixl1	TGCTCCTGGGACTGAAGCTA	CAACGCTCCCAGAATCCTCA
Pyy*	TAATCCTGCTCGCCTGTCTG	TTCACCACTGGTCCAAACCT
Slc22a2	AGAGTCGTCCTGAGCTGACC	AACTCAGTGACGATGGAGGAG
Slc22a3	ACTCACTGCCTTCCCCAAC	AGAGATGGCTAGCTGGATGC
Sox17	GGAGGGAGGGTCACCACTGCT	CTATGGCCACGGGACACGCC
Sox7	CTGTCCGGATGGGCTGTCGCC	TTCTTCCTGCGGGGCCGGTA
Tfpi2	TTTGCCGCTGCTCCTGGTGG	TGGCATTGCCCAAGCAGCCT
Ttr*	AGACCTCTGAGGGATCCTGG	ATGGGATGCTACTGCTTTGG
Primers used for Sanger sequencing RT-PCR amplicons (Fig.2C,D; Fig.S2)		
**Gene**	**Forward**	**Reverse**
Airn	TGCAGAAAAATGGCTCTGTTCTTA	ACCGTAACAGTTTCACCTACGA
Cd81	AGCTGTCATTATGATCTTTGAGATG	AATGGAGGCAGGAAAGGAACA
Cdknc1	CAGGGTGTCCCTCTCCAAAC	TTTACACCTTGGGACCAGCG
Igf2	GGGGGTGTCAATTGGGTTGT	CCTCTTCGGAGATGTCCAGC
Igf2r	TCAGCAGGAAAGTAGGCACC	GACTCCGCTCTGAGAGTCCT
Kcnq1ot1	CCAGACCCGATTCGGTTTCA	GGCCAGAAGCAGAGGTGATT
Osbpl5	TTCTCTCTACGGACTGCCCA	GAAGTCTCGTCCAGCTCTCC
Peg10	TCCACCCATTTGTCTGCCAT	GGCCTGCCTCTTTCATCTCC
Pon2	CTCAAGTTTCCAGGGCTCCA	CATCTATGAAAGTGCTAATGCCATGT
Ppp1r9a	AAGTCTGCCCTGCCTGTAAC	TTTGTTTCCACACCGGACCA
Sfmbt2 (Cast x FVB)	CGCAGAGGAGGACGTGTTGCC	CTGGAGGGCGCTGGCACTTC
Sfmbt2 (Bl6 x 129)	AATGGAAATGCCACCTCAAC	CGTCTGAAGATGGCAGTGAA
Slc22a3 (Cast x FVB, Bl6 x 129)	ACTCACTGCCTTCCCCAAC	AGAGATGGCTAGCTGGATGC
Tssc4	GAGGCTGAGGAATGGAGTGG	TCCTAAACACTGGGGCACAA

Table S4. Samples sequenced by RNA-seq, WGBS and ChIP-seq.

**RNA-seq**	Sample information					
Sample	Stage	Genotype	Sex	Seq. depth (reads)	Read type	Library prep. Method
Yolk sac endoderm (br.1)	E12.5	FVB/N	M+F	77,019,807	51bp Single End	(Huang et al 2011)
Yolk sac endoderm (br.2)*	E12.5	F1 CAST/FVB	M+F	50,858,688	101bp Paired End	(Sultan et al 2012)
Yolk sac endoderm (br.3)*	E12.5	F1 CAST/FVB	M+F	53,269,491	101bp Paired End	(Sultan et al 2012)
Yolk sac endoderm (br.4)*	E12.5	F1 CAST/FVB	M+F	58,680,944	101bp Paired End	(Sultan et al 2012)
Yolk sac endoderm (br.5)*	E12.5	F1 CAST/FVB	M+F	53,202,468	101bp Paired End	(Sultan et al 2012)
Yolk sac endoderm (br.1)	E9.25	FVB/N	M+F	63,805,819	101bp Paired End	(Sultan et al 2012)
Yolk sac endoderm (br.2)	E9.25	FVB/N	M+F	103,262,624	101bp Paired End	(Sultan et al 2012)
Yolk sac mesoderm (br.1)	E9.25	FVB/N	M+F	69,322,057	101bp Paired End	(Sultan et al 2012)
Yolk sac mesoderm (br.2)	E9.25	FVB/N	M+F	71,521,422	101bp Paired End	(Sultan et al 2012)
Fetal liver (br.1)*	E12.5	F1 CAST/FVB	M+F	51,077,974	101bp Paired End	(Sultan et al 2012)
Fetal liver (br.2)*	E12.5	F1 CAST/FVB	M+F	56,585,937	101bp Paired End	(Sultan et al 2012)
Fetal liver (br.3)*	E12.5	F1 CAST/FVB	M+F	52,303,488	101bp Paired End	(Sultan et al 2012)
Fetal liver (br.4)*	E12.5	F1 CAST/FVB	M+F	52,061,503	101bp Paired End	(Sultan et al 2012)
Fetal liver (br.5)	E12.5	FVB/N	M+F	67,562,979	101bp Paired End	ScriptSeq v1
Fetal liver (br.6)	E12.5	FVB/N	M+F	48,766,733	101bp Paired End	ScriptSeq v1
Cystic EBs (A9, br.1)	d10	C57BL/6 x 129	F	126,715,112	51bp Single End	ScriptSeq v1
Cystic EBs (JN, br.2)	d10	C57BL/6 x 129	M	61,201,370	101bp Paired End	(Sultan et al 2012)
Cystic EBs (A9, br.1)	d15	C57BL/6 x 129	F	122,826,880	51bp Single End	ScriptSeq v1
Cystic EBs (JN, br.2)	d15	C57BL/6 x 129	M	68,175,994	101bp Paired End	(Sultan et al 2012)
**WGBS**	Sample information					
Sample	Stage	Genotype	Sex	Seq. depth (reads)	Read type	Library prep. Method
Yolk sac endoderm	E12.5	CAST/EiJ x FVB/N	M+F	152,951,808	101bp Paired End	TruSeq v2
Yolk sac endoderm	E12.5	FVB/N x CAST/EiJ	M+F	56,573,815	101bp Paired End	TruSeq v2
Fetal liver	E12.5	CAST/EiJ x FVB/N	M+F	88,378,228	101bp Paired End	TruSeq v2
Fetal liver	E12.5	FVB/N x CAST/EiJ	M+F	87,382,218	101bp Paired End	TruSeq v2
Cystic EBs (CF-C2)	d15	CAST/EiJ x FVB/N	M	83,240,691	101bp Paired End	TruSeq v2
Cystic EBs (FC-A2)	d15	FVB/N x CAST/EiJ	M	41,643,829	101bp Paired End	TruSeq v2
**ChIP-seq**	Sample information					
Sample	Stage	Genotype	Sex	Seq. depth (reads)	Read type	Library prep. Method
VYS H3K4me3 ChIP	E12.5	FVB/N	M+F	13,858,725	51bp Single End	TruSeq ChIP
VYS input	E12.5	FVB/N	M+F	30,163,909	51bp Single End	TruSeq ChIP

Maternal allele written on left except for E12.5 ysE and fetal liver F1 CAST/FVB crosses for RNA-seq (*) where the direction of the cross is not indicated.

Sequences submitted to GEO (GSE56276) except for extra biological replicates (*) that will submitted together with a manuscript currently in preparation (DA & QJH et al) surveying imprinted expression in multiple tissues.

Key: Sample: br = biological replicate, ES cell clone name specified. Sex = male (M), female (F).
